# Supplementary figures and images for: ParB spreading on DNA requires cytidine triphosphate in vitro
Source: eLife. 2020 Feb 20;9:e53515. doi: 10.7554/eLife.53515 (PMC7053999; doi:10.7554/eLife.53515)

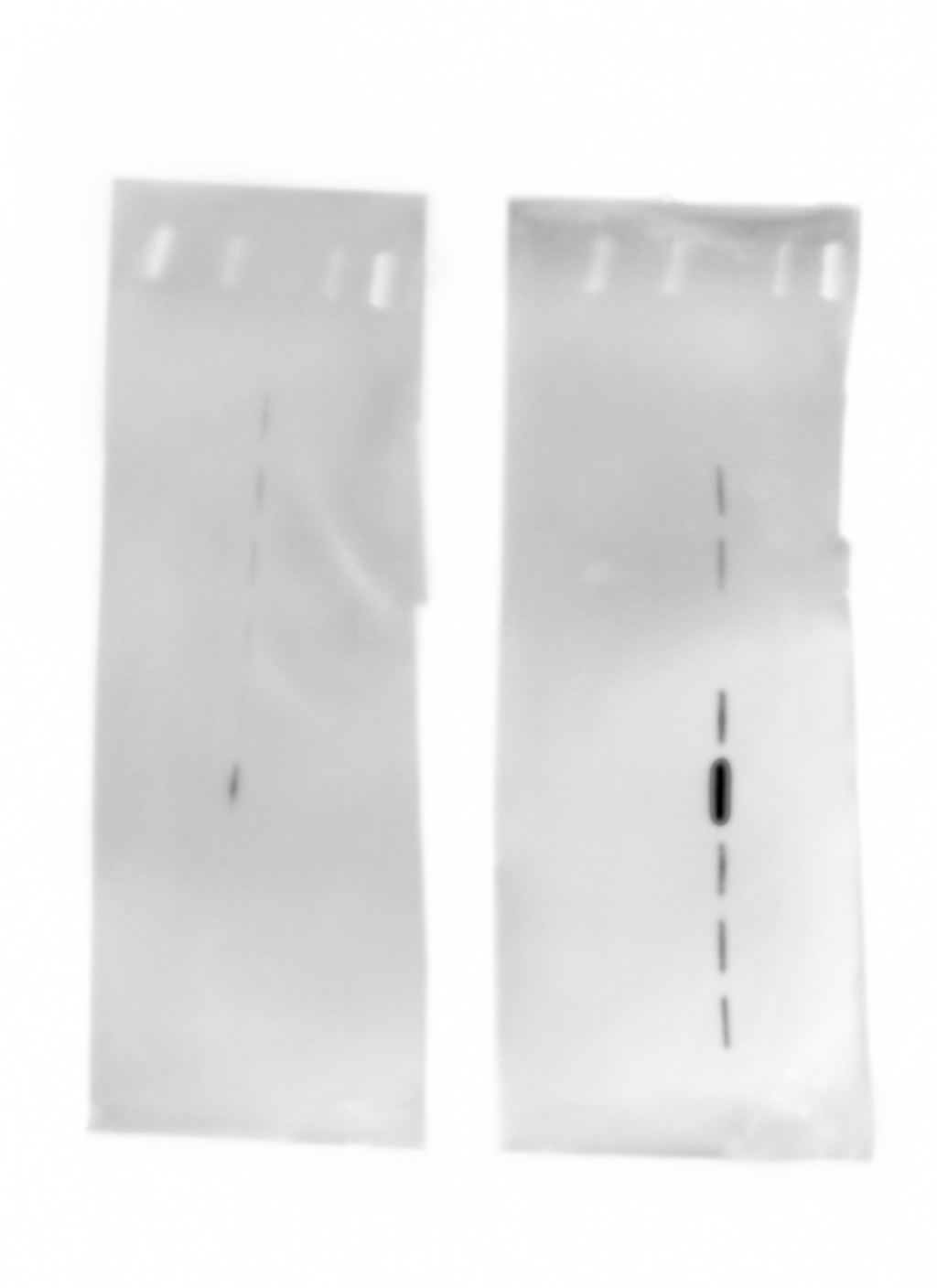

Supplement: Figure 2—source data 1. [file elife-53515-fig2-data1.zip › Source_data_Figure2/Figure2_panelC/2019.10.16_first_Western_blot_repeat_Fig2C_and_3D_all_in_one/2019.10.16_13.34.36_Ch.tif]

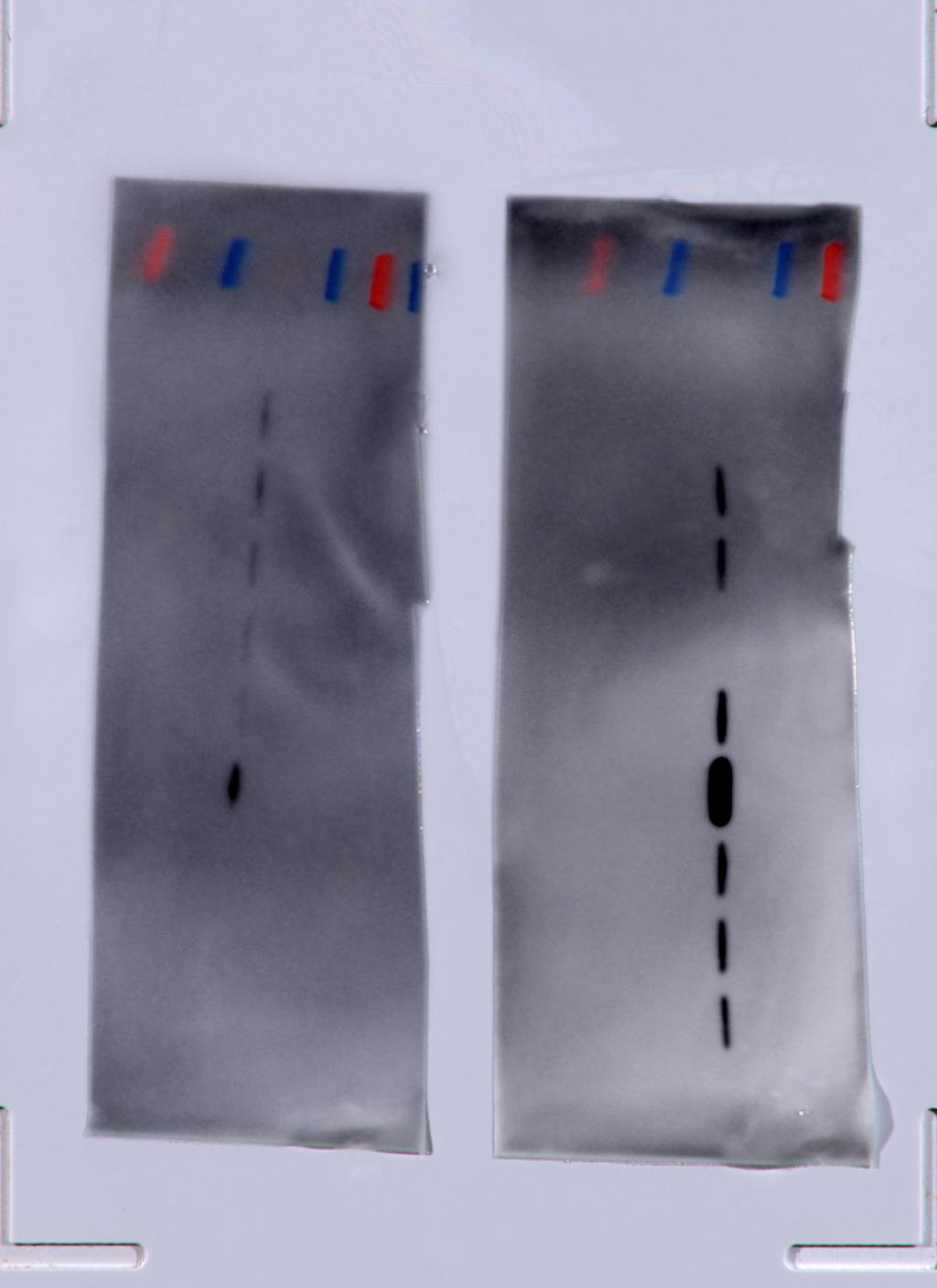

Supplement: Figure 2—source data 1. [file elife-53515-fig2-data1.zip › Source_data_Figure2/Figure2_panelC/2019.10.16_first_Western_blot_repeat_Fig2C_and_3D_all_in_one/2019.10.16_13.34.36_Ch+Marker.jpg]

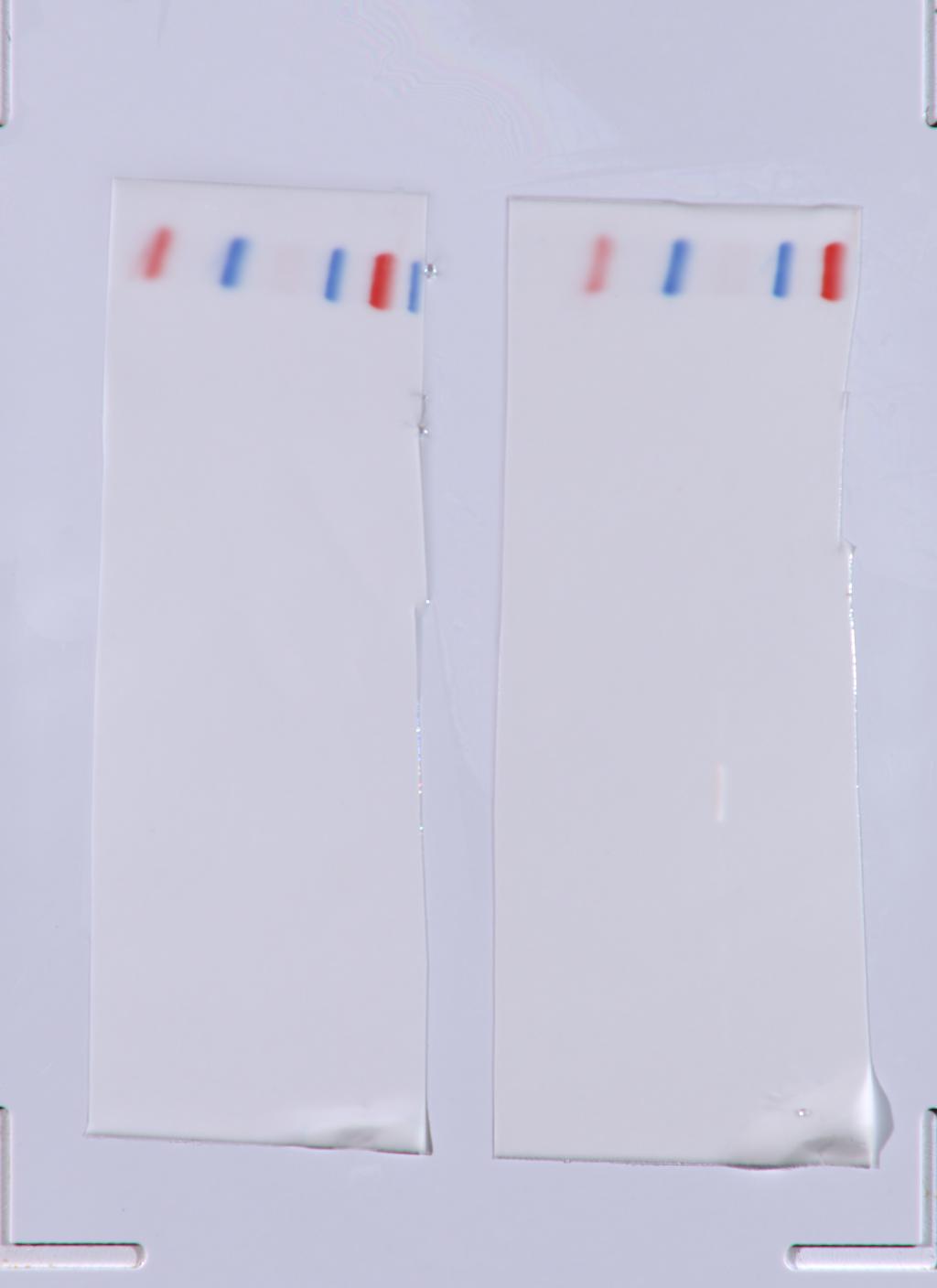

Supplement: Figure 2—source data 1. [file elife-53515-fig2-data1.zip › Source_data_Figure2/Figure2_panelC/2019.10.16_first_Western_blot_repeat_Fig2C_and_3D_all_in_one/2019.10.16_13.34.36_Ch-Marker.jpg]

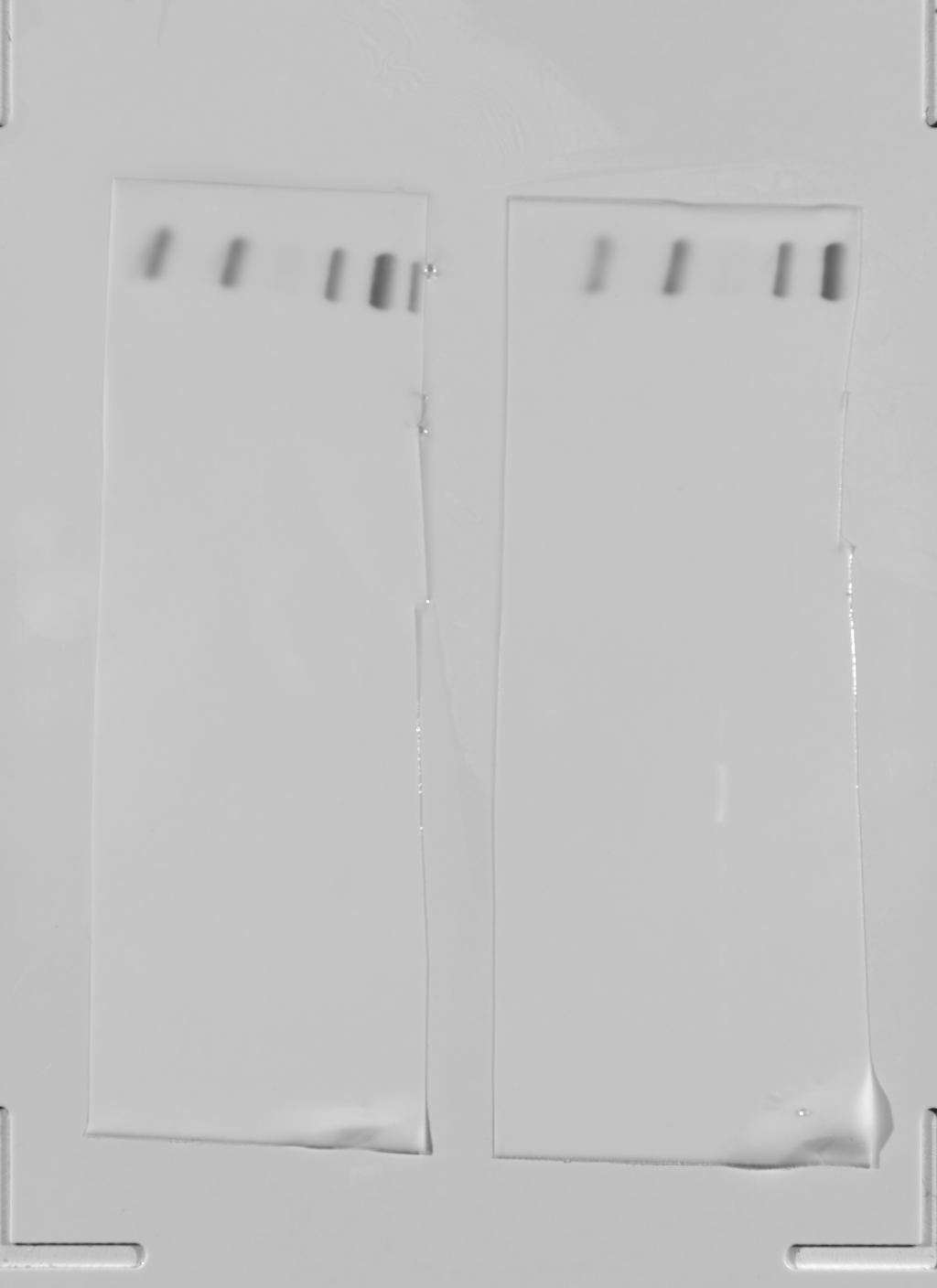

Supplement: Figure 2—source data 1. [file elife-53515-fig2-data1.zip › Source_data_Figure2/Figure2_panelC/2019.10.16_first_Western_blot_repeat_Fig2C_and_3D_all_in_one/2019.10.16_13.34.36_Ch-Marker.tif]

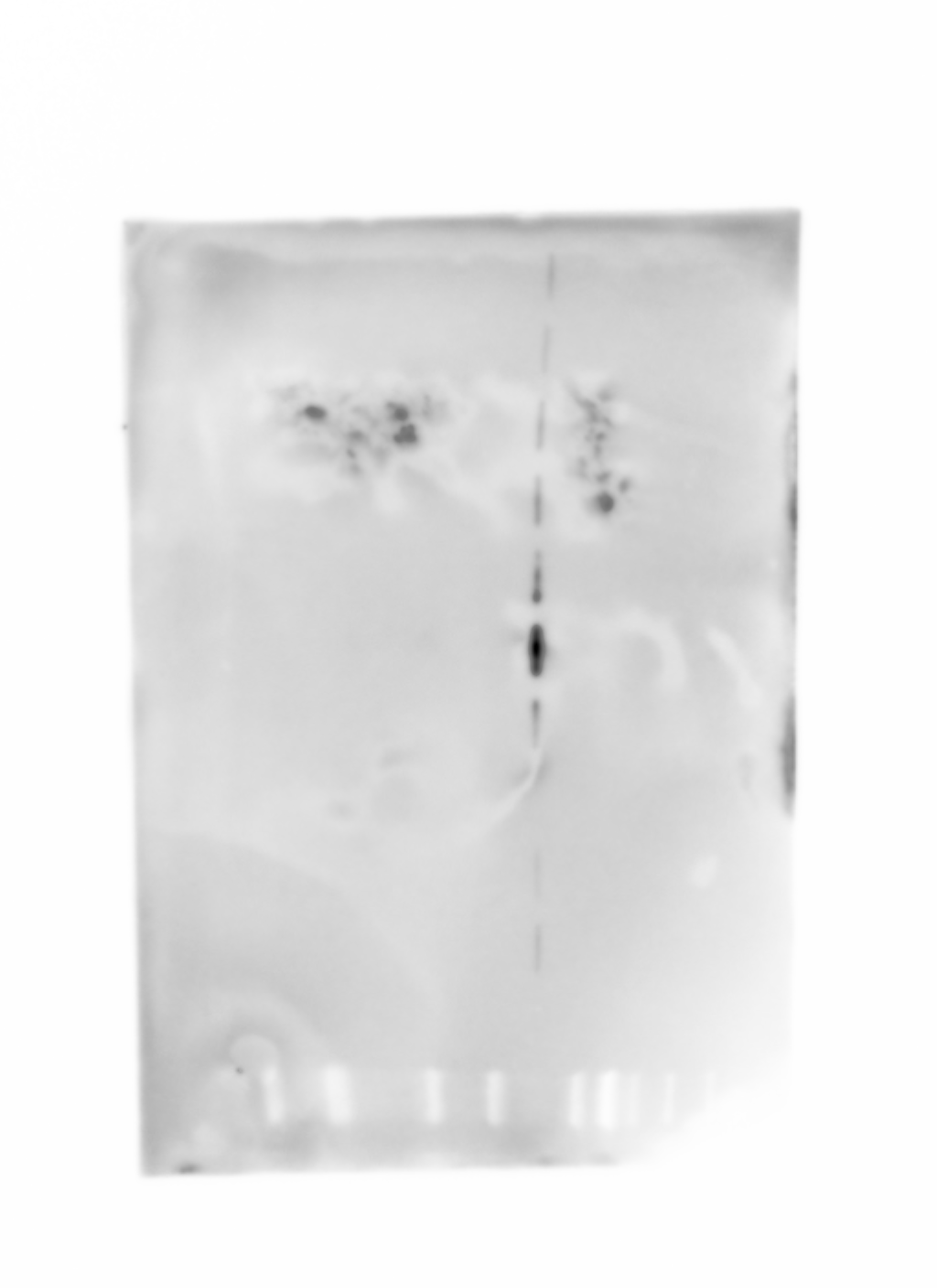

Supplement: Figure 2—source data 1. [file elife-53515-fig2-data1.zip › Source_data_Figure2/Figure2_panelC/2019.10.18_13.47.50_Ch_Fig2C_repeats/2019.10.18_13.47.50_Ch.tif]

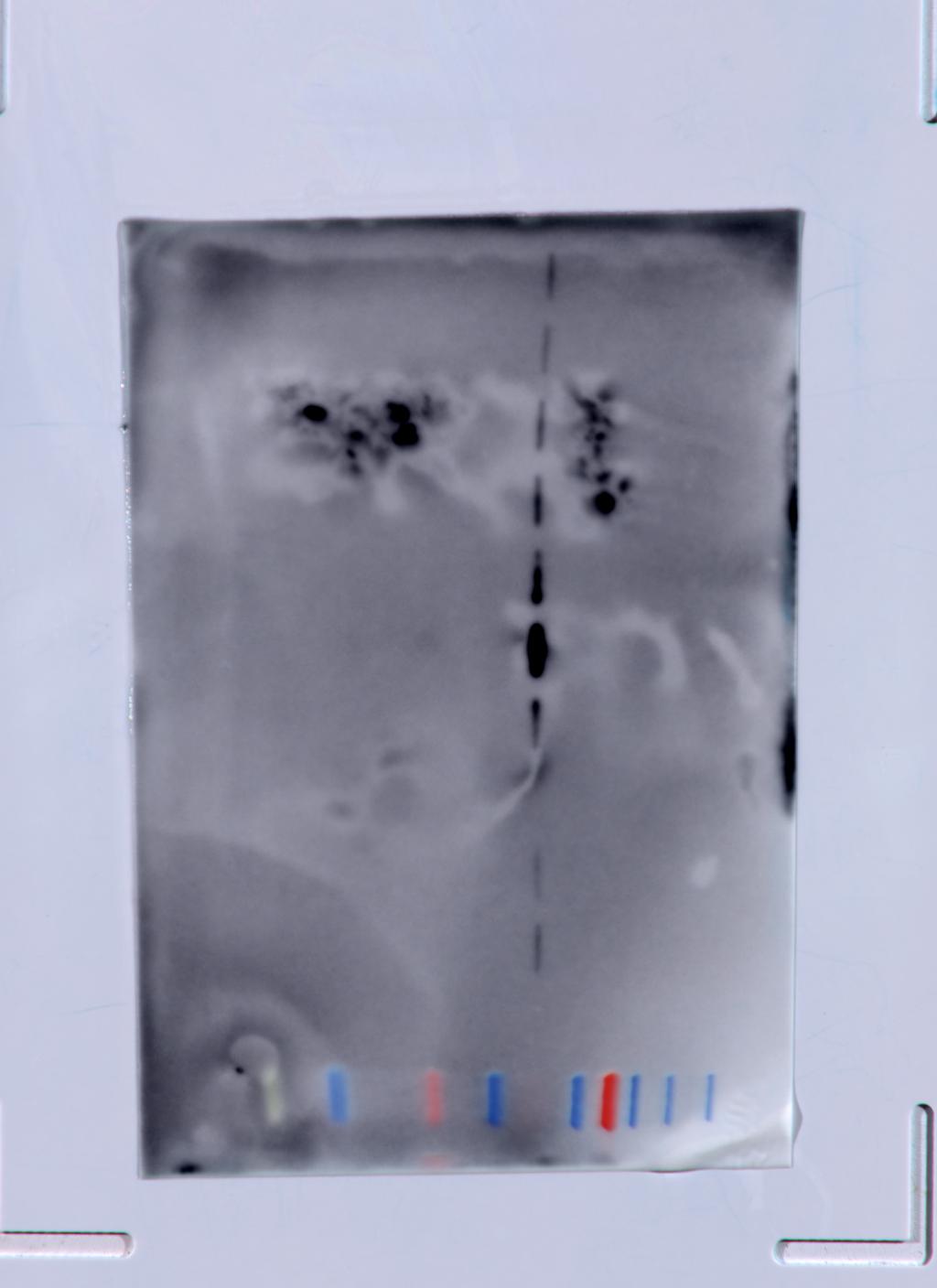

Supplement: Figure 2—source data 1. [file elife-53515-fig2-data1.zip › Source_data_Figure2/Figure2_panelC/2019.10.18_13.47.50_Ch_Fig2C_repeats/2019.10.18_13.47.50_Ch+Marker.jpg]

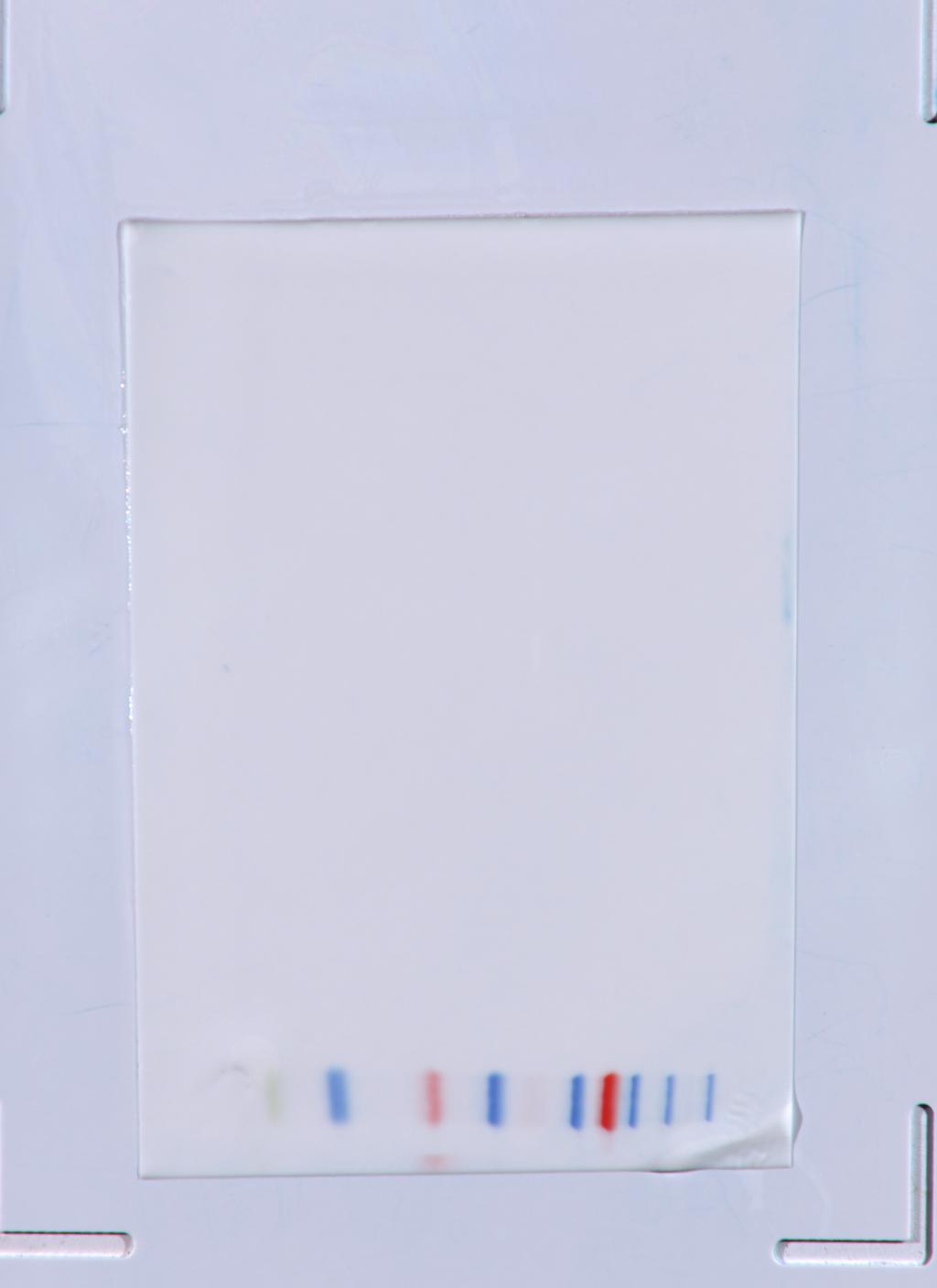

Supplement: Figure 2—source data 1. [file elife-53515-fig2-data1.zip › Source_data_Figure2/Figure2_panelC/2019.10.18_13.47.50_Ch_Fig2C_repeats/2019.10.18_13.47.50_Ch-Marker.jpg]

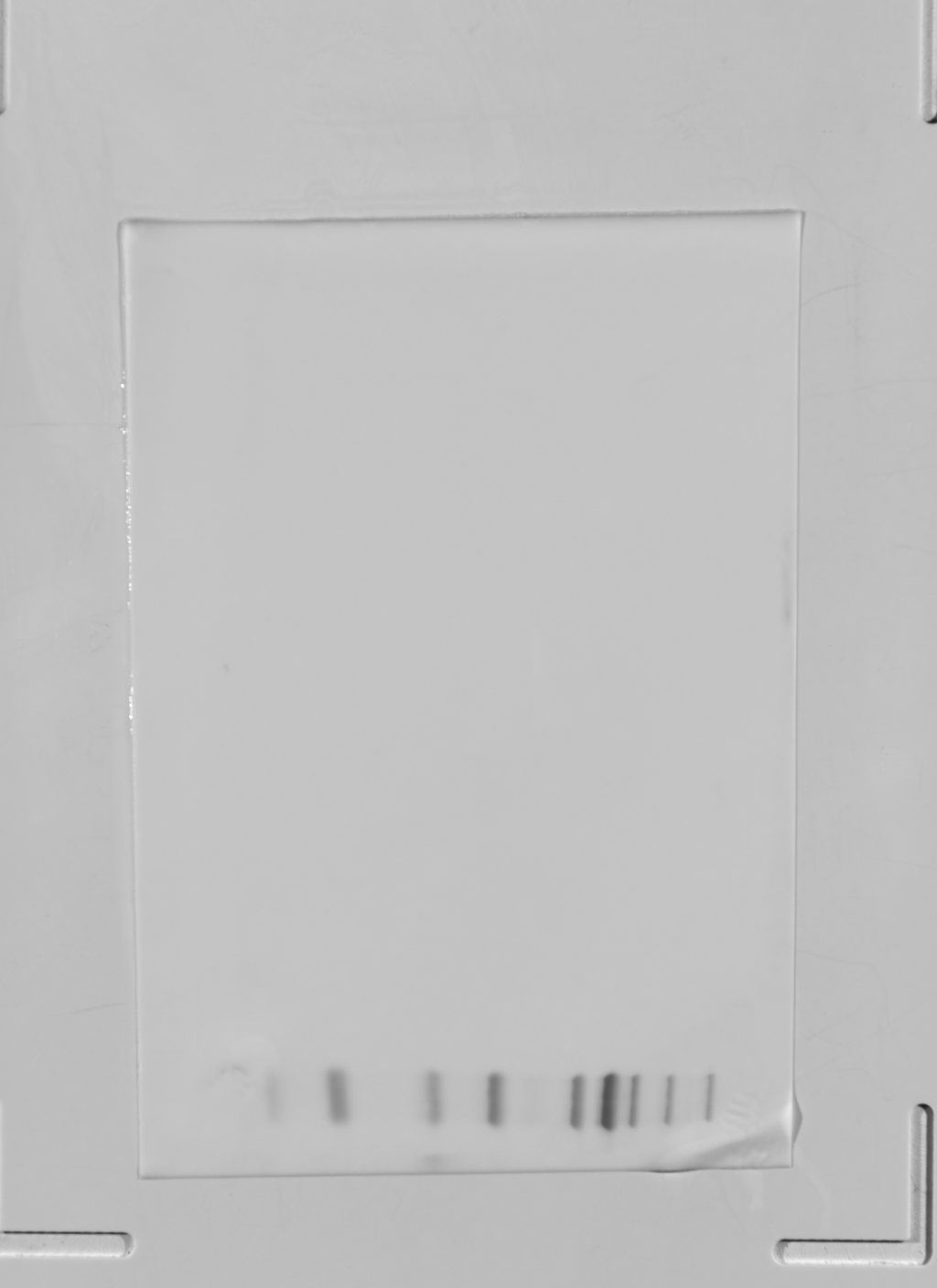

Supplement: Figure 2—source data 1. [file elife-53515-fig2-data1.zip › Source_data_Figure2/Figure2_panelC/2019.10.18_13.47.50_Ch_Fig2C_repeats/2019.10.18_13.47.50_Ch-Marker.tif]

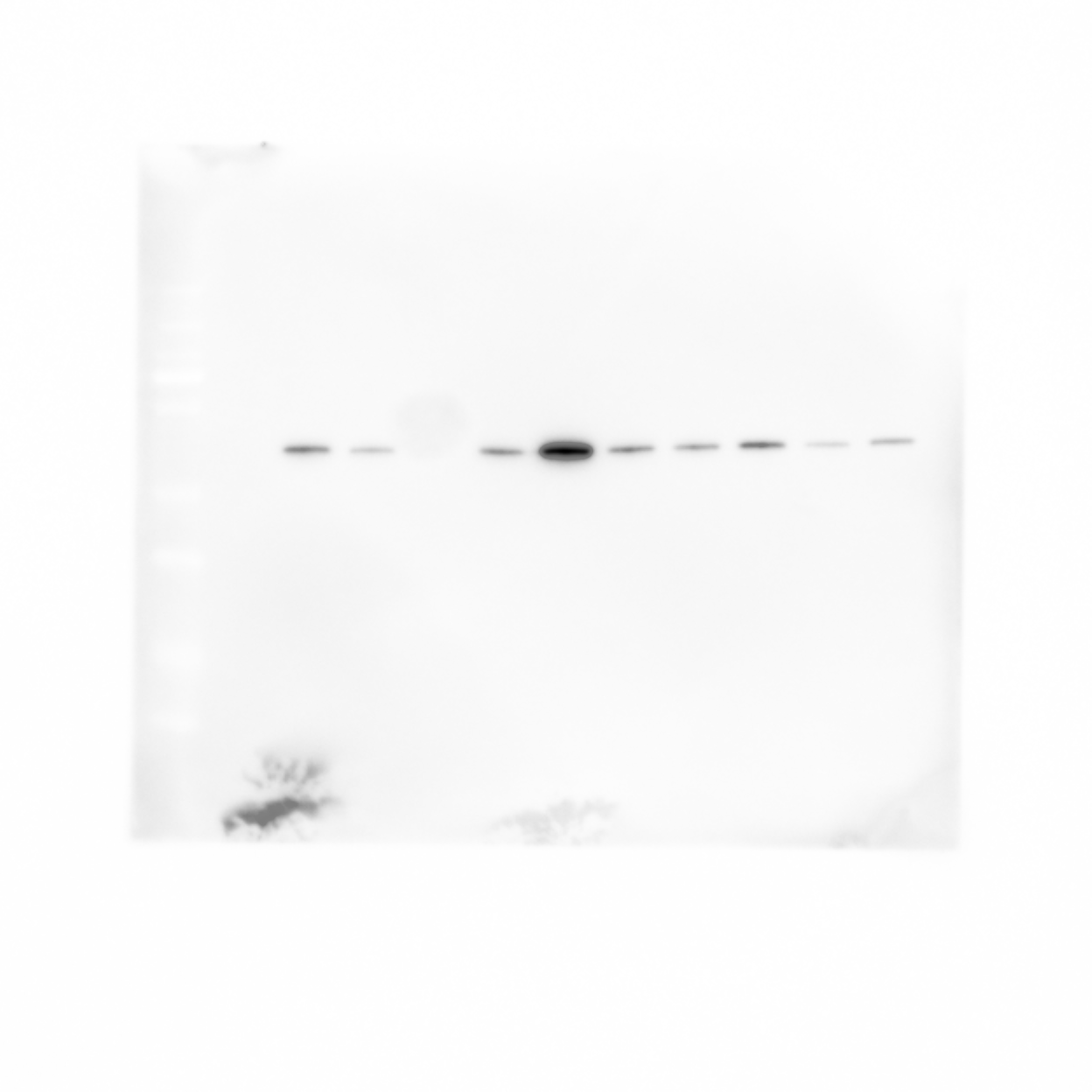

Supplement: Figure 2—source data 1. [file elife-53515-fig2-data1.zip › Source_data_Figure2/Figure2_panelC/20191101_145408_Ch_Fig2C_in_manuscript/20191101_145408_Ch.tif]

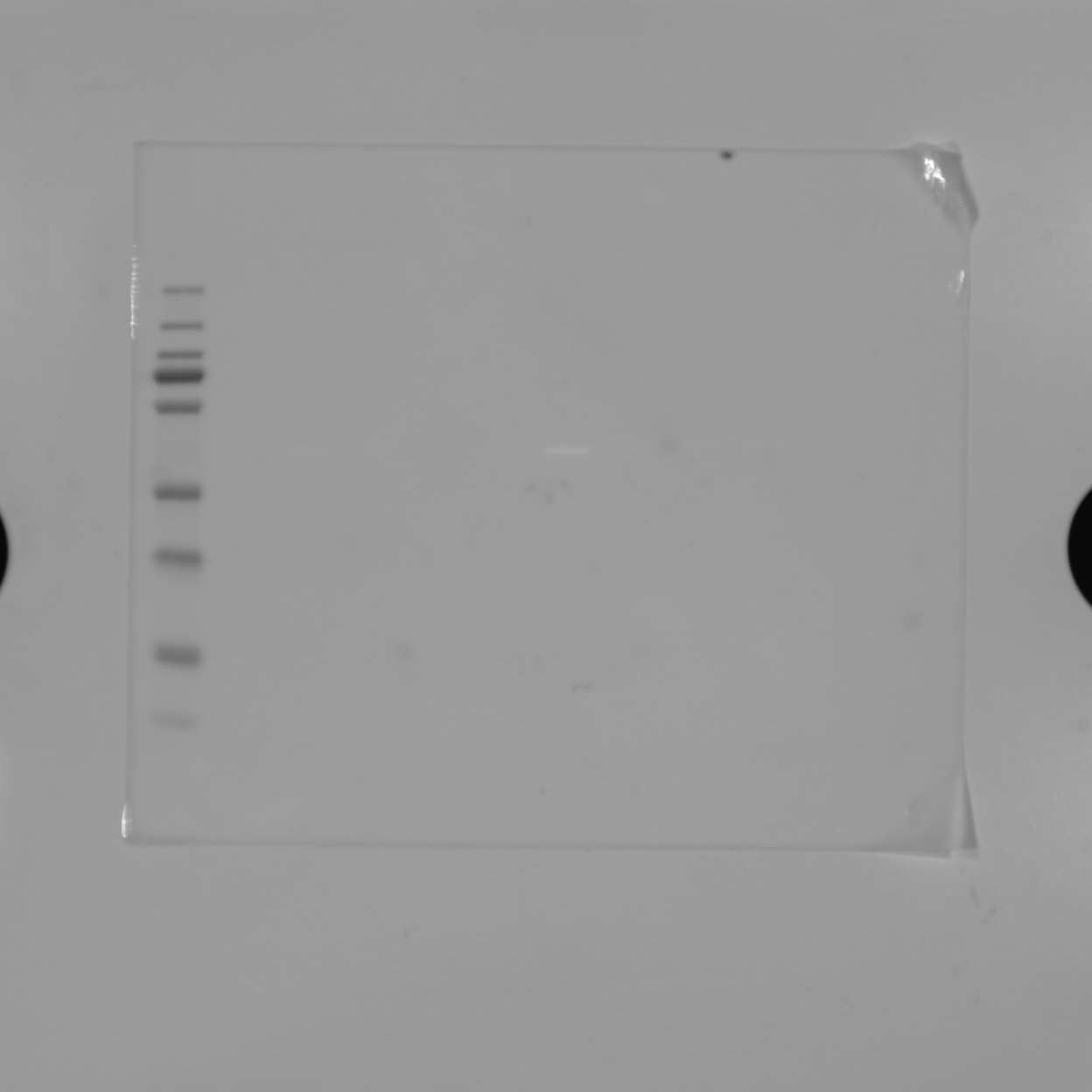

Supplement: Figure 2—source data 1. [file elife-53515-fig2-data1.zip › Source_data_Figure2/Figure2_panelC/20191101_145408_Ch_Fig2C_in_manuscript/20191101_145408_Ch_Marker.tif]

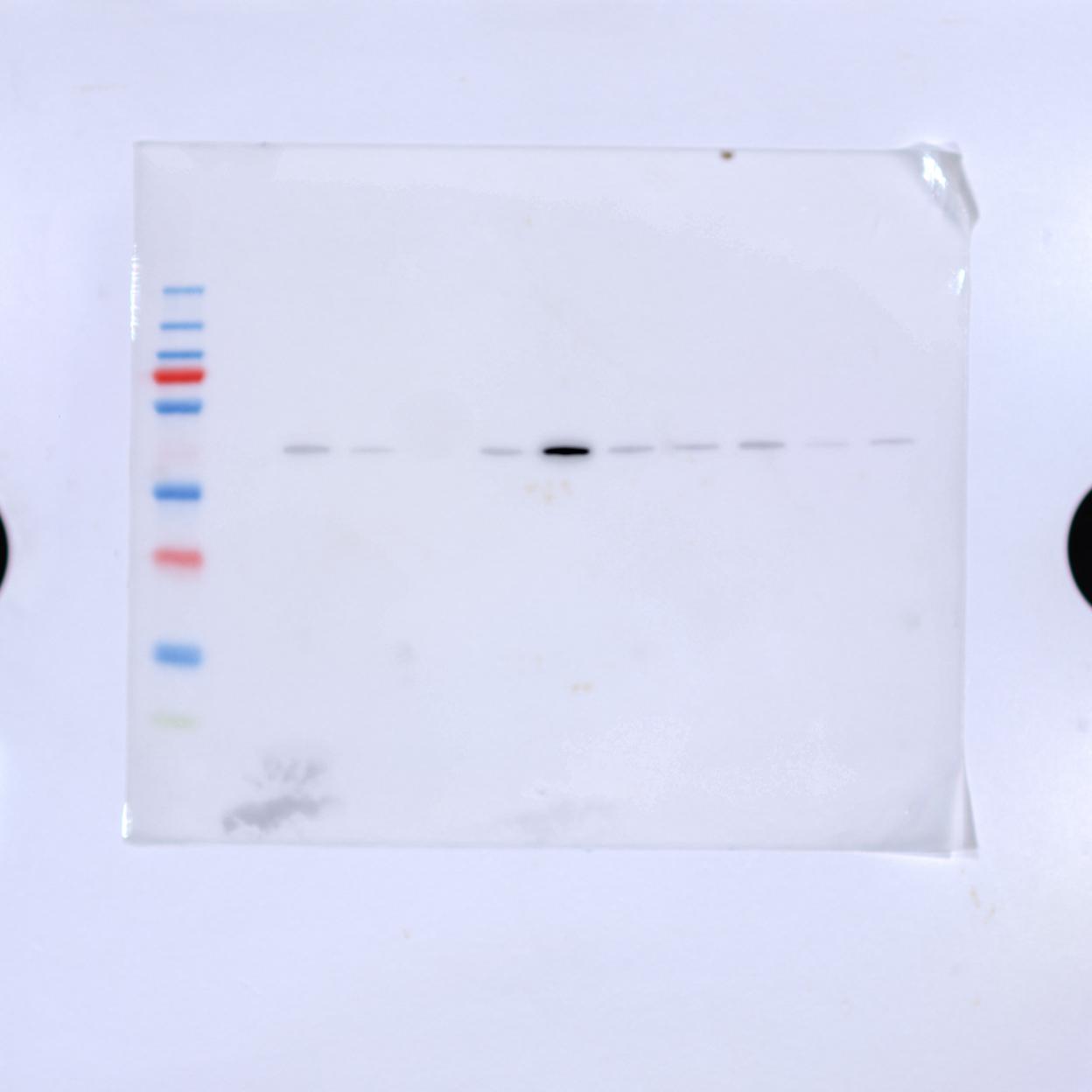

Supplement: Figure 2—source data 1. [file elife-53515-fig2-data1.zip › Source_data_Figure2/Figure2_panelC/20191101_145408_Ch_Fig2C_in_manuscript/20191101_145408_Ch+Marker.jpg]

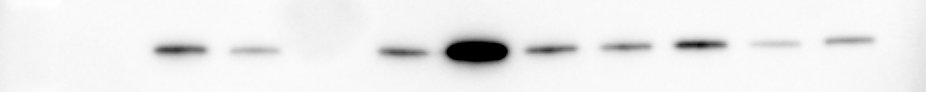

Supplement: Figure 2—source data 1. [file elife-53515-fig2-data1.zip › Source_data_Figure2/Figure2_panelC/20191101_145408_Ch_Fig2C_in_manuscript/Cropped_images/20191101_145408_Ch-1.jpg]

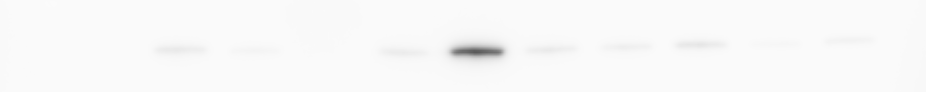

Supplement: Figure 2—source data 1. [file elife-53515-fig2-data1.zip › Source_data_Figure2/Figure2_panelC/20191101_145408_Ch_Fig2C_in_manuscript/Cropped_images/20191101_145408_Ch-1.tif]

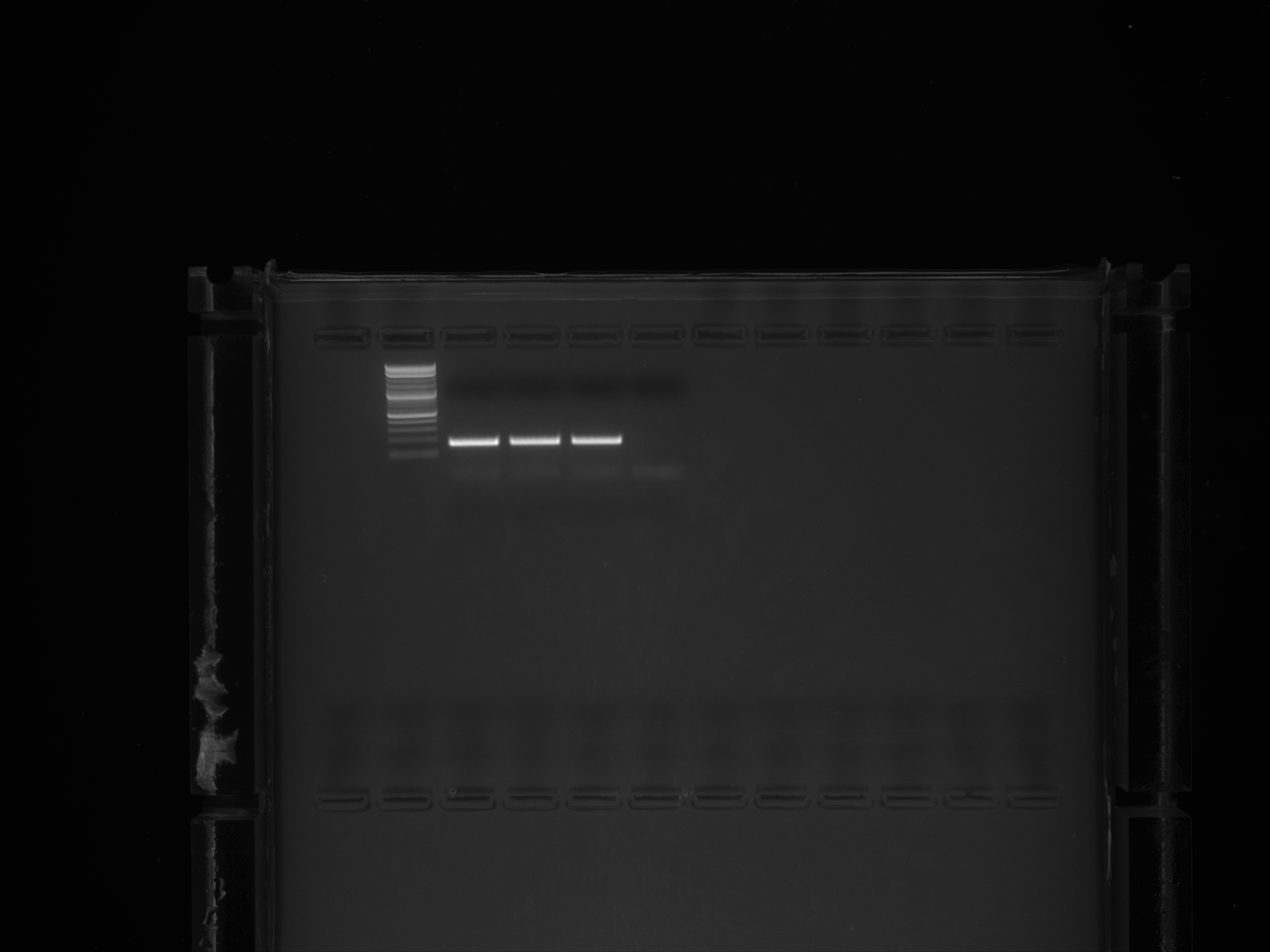

Supplement: Figure 2—figure supplement 1—source data 1. [file elife-53515-fig2-figsupp1-data1.zip › Source_data_Figure2_figure_supplement1/EcoT7_ecoVII_doubel_treatment_in_manuscript_uncropped.Tif]

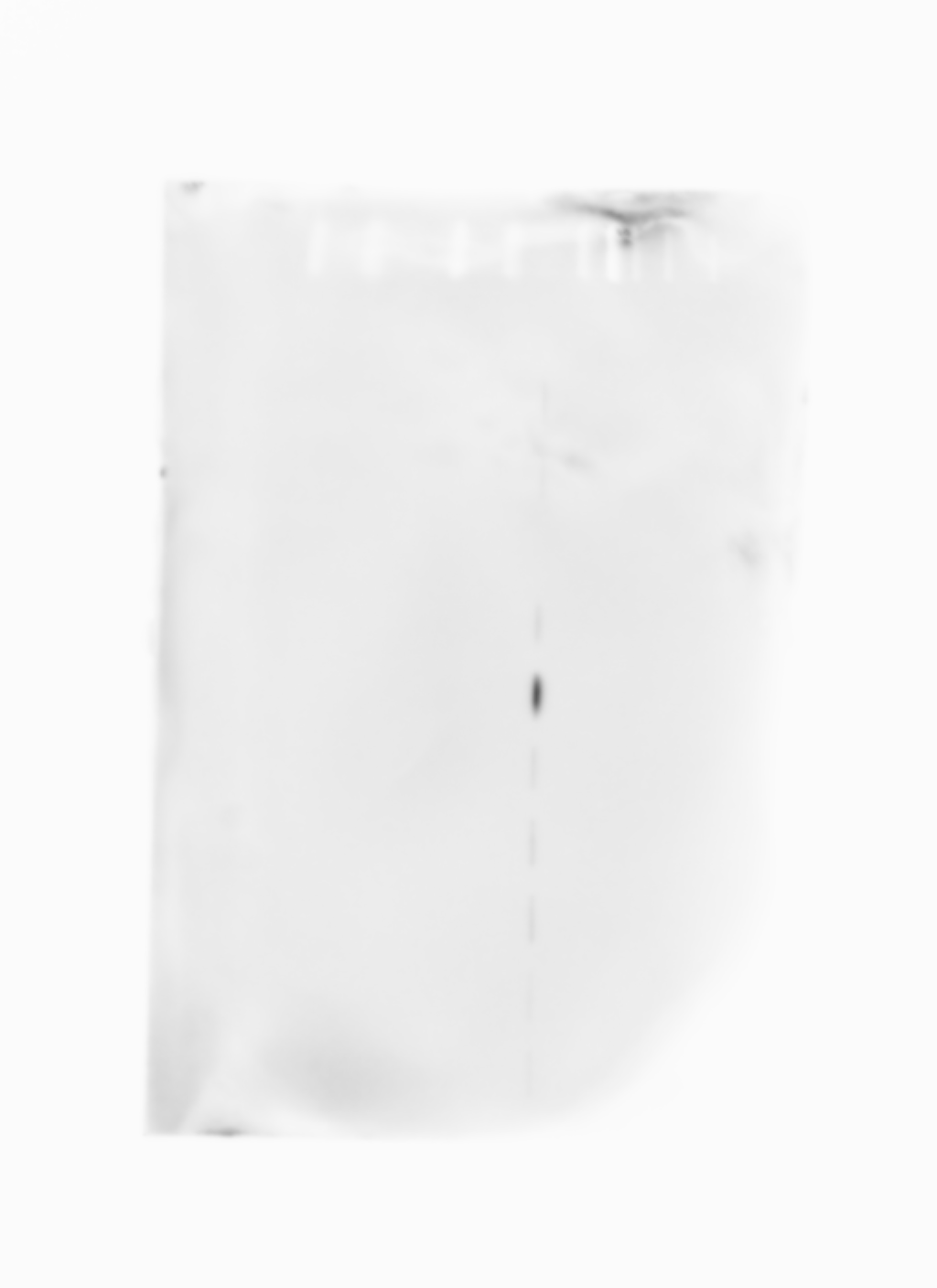

Supplement: Figure 3—source data 1. [file elife-53515-fig3-data1.zip › Source_data_Figure3/Figure3_panelD/2019.10.18_13.37.02_Ch_Fig3D_repeats/2019.10.18_13.37.02_Ch.tif]

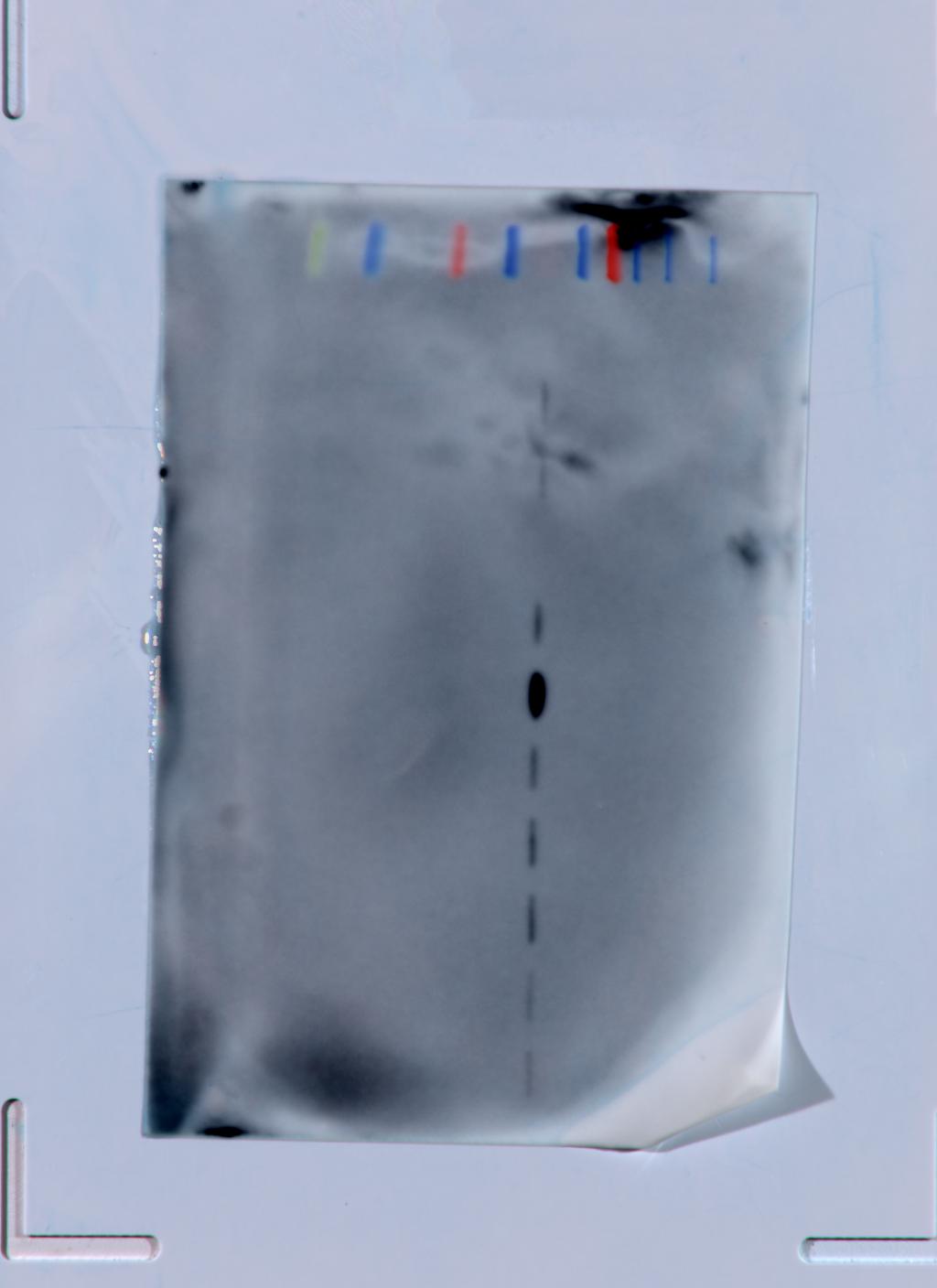

Supplement: Figure 3—source data 1. [file elife-53515-fig3-data1.zip › Source_data_Figure3/Figure3_panelD/2019.10.18_13.37.02_Ch_Fig3D_repeats/2019.10.18_13.37.02_Ch+Marker.jpg]

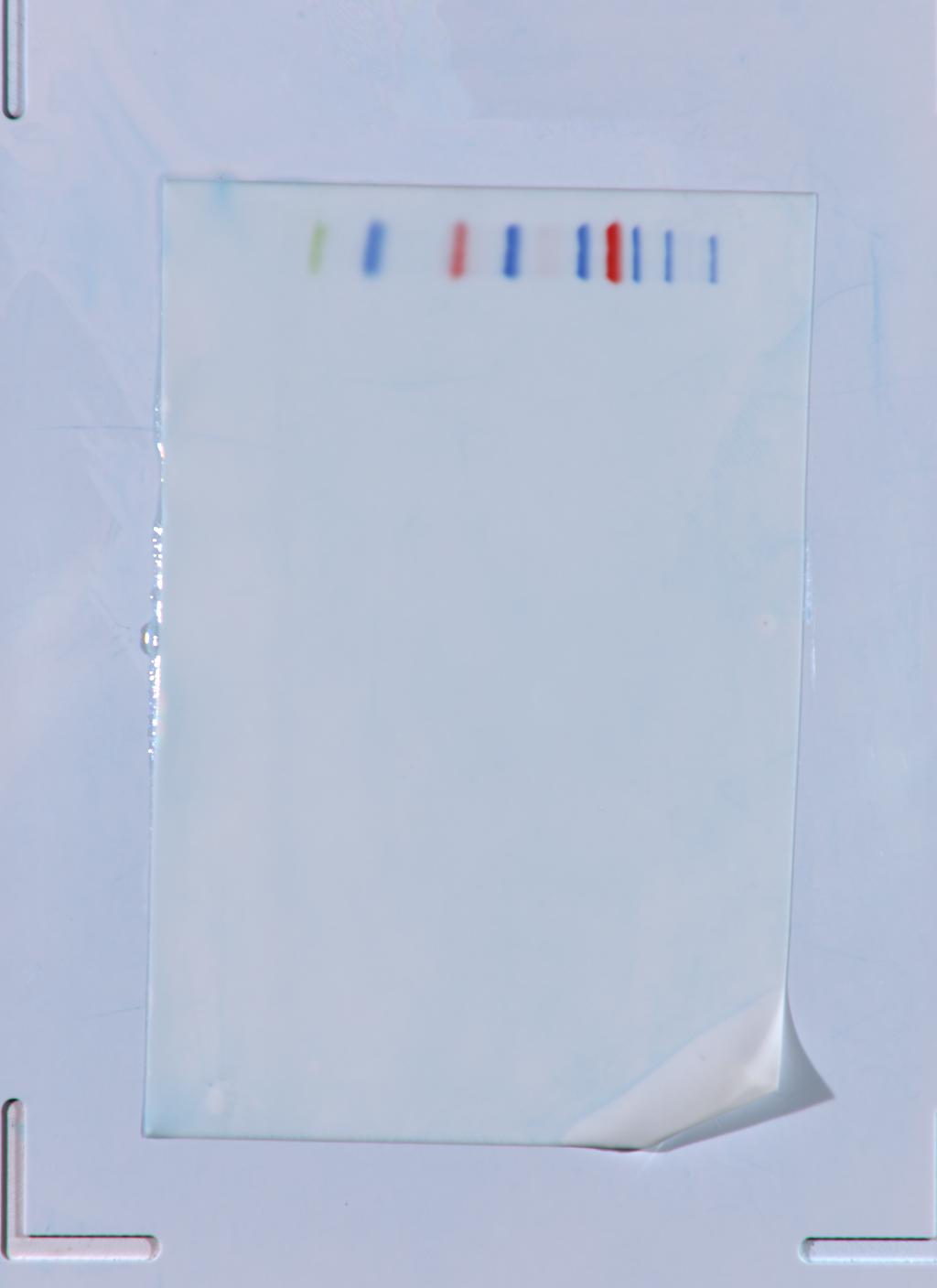

Supplement: Figure 3—source data 1. [file elife-53515-fig3-data1.zip › Source_data_Figure3/Figure3_panelD/2019.10.18_13.37.02_Ch_Fig3D_repeats/2019.10.18_13.37.02_Ch-Marker.jpg]

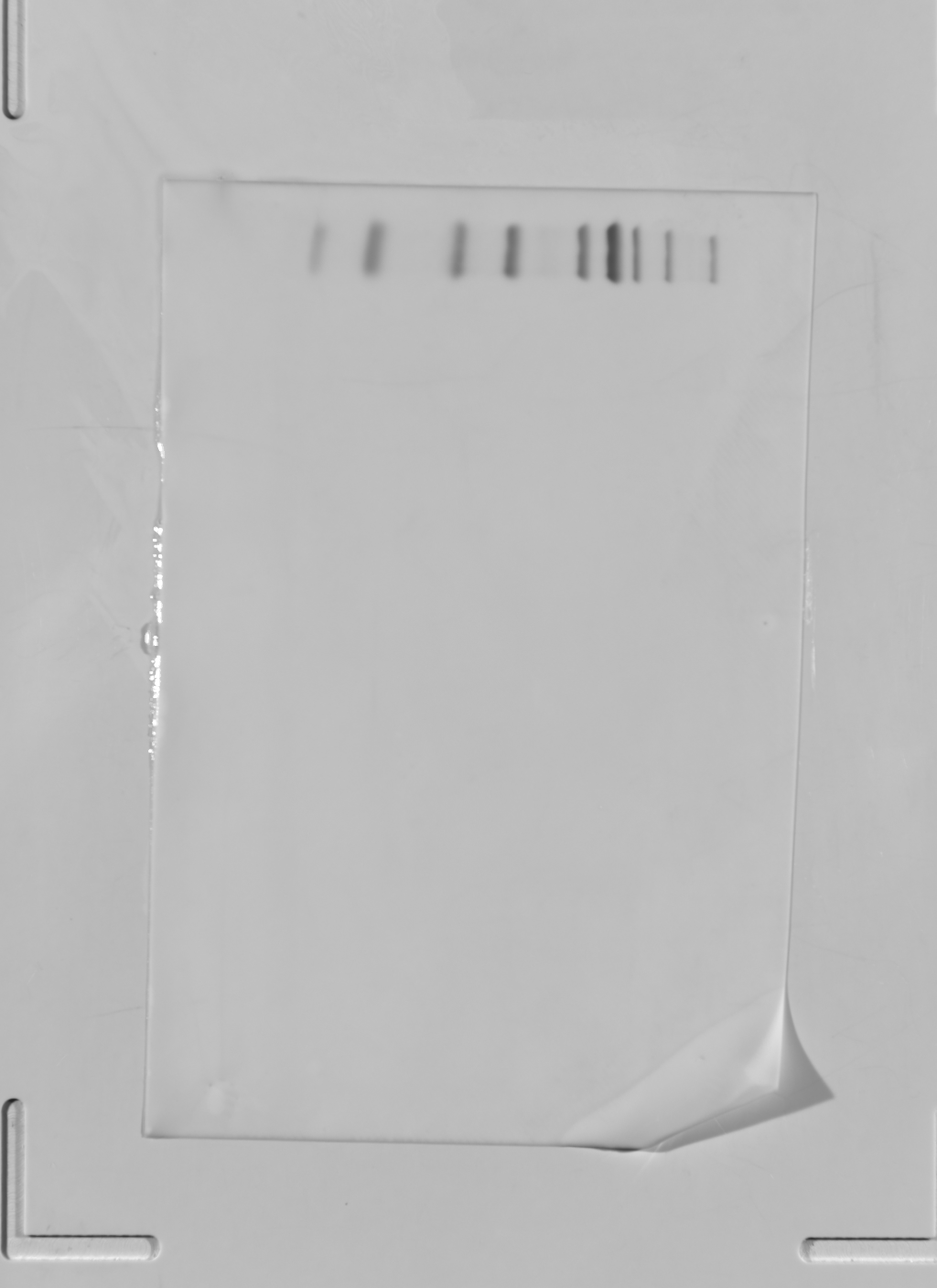

Supplement: Figure 3—source data 1. [file elife-53515-fig3-data1.zip › Source_data_Figure3/Figure3_panelD/2019.10.18_13.37.02_Ch_Fig3D_repeats/2019.10.18_13.37.02_Ch-Marker.tif]

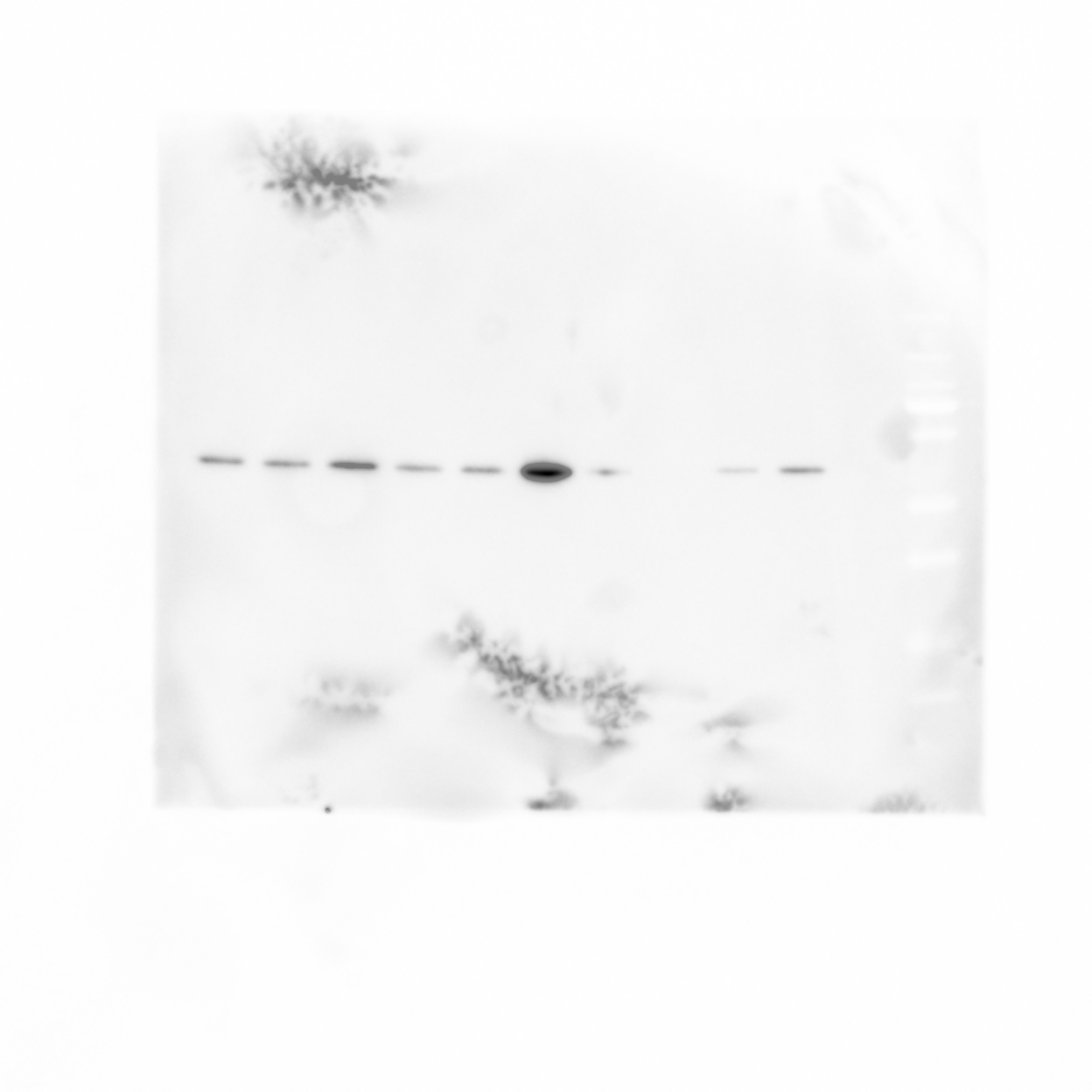

Supplement: Figure 3—source data 1. [file elife-53515-fig3-data1.zip › Source_data_Figure3/Figure3_panelD/20191101_145700_Ch_Fig3D_in_manuscript/20191101_145700_Ch.tif]

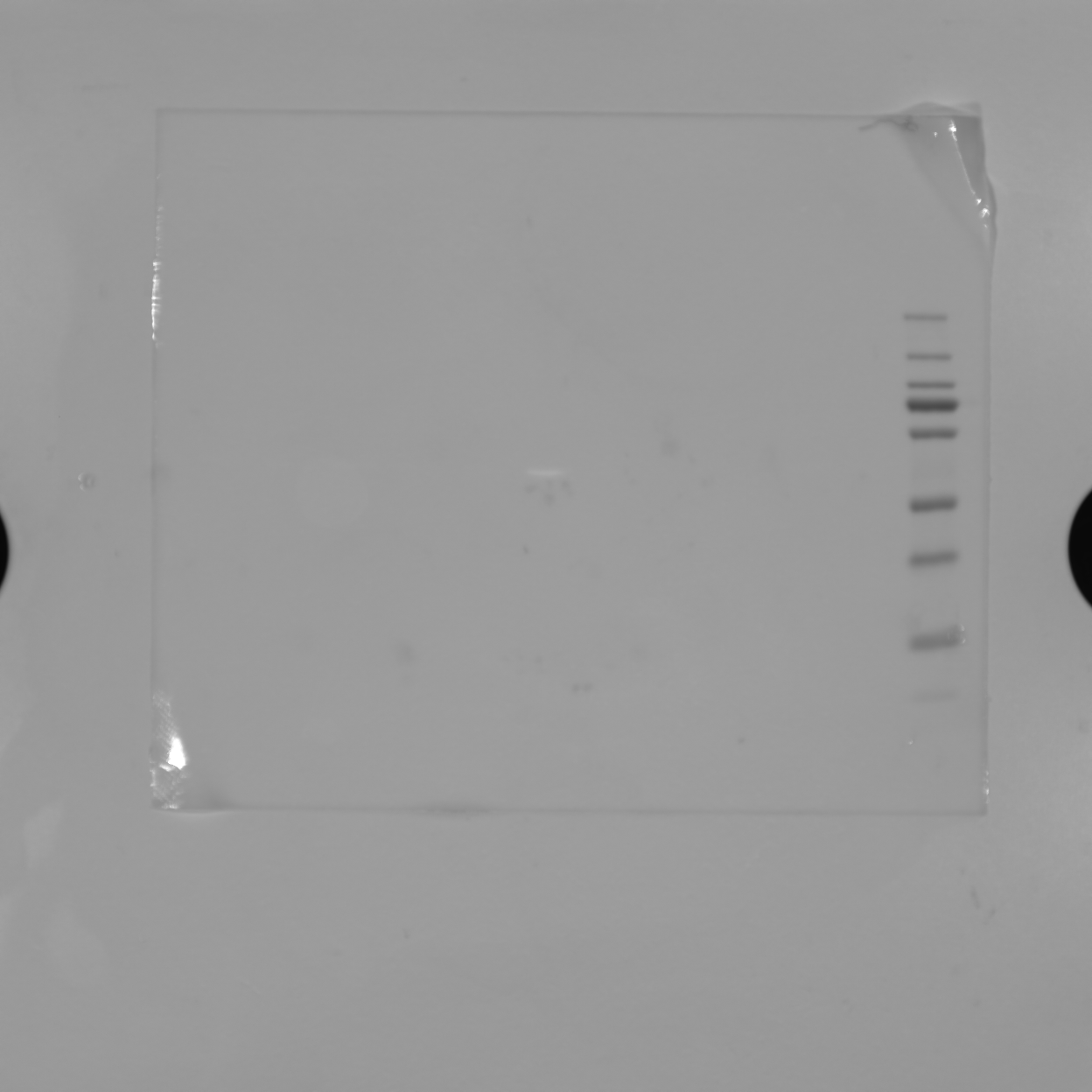

Supplement: Figure 3—source data 1. [file elife-53515-fig3-data1.zip › Source_data_Figure3/Figure3_panelD/20191101_145700_Ch_Fig3D_in_manuscript/20191101_145700_Ch_Marker.tif]

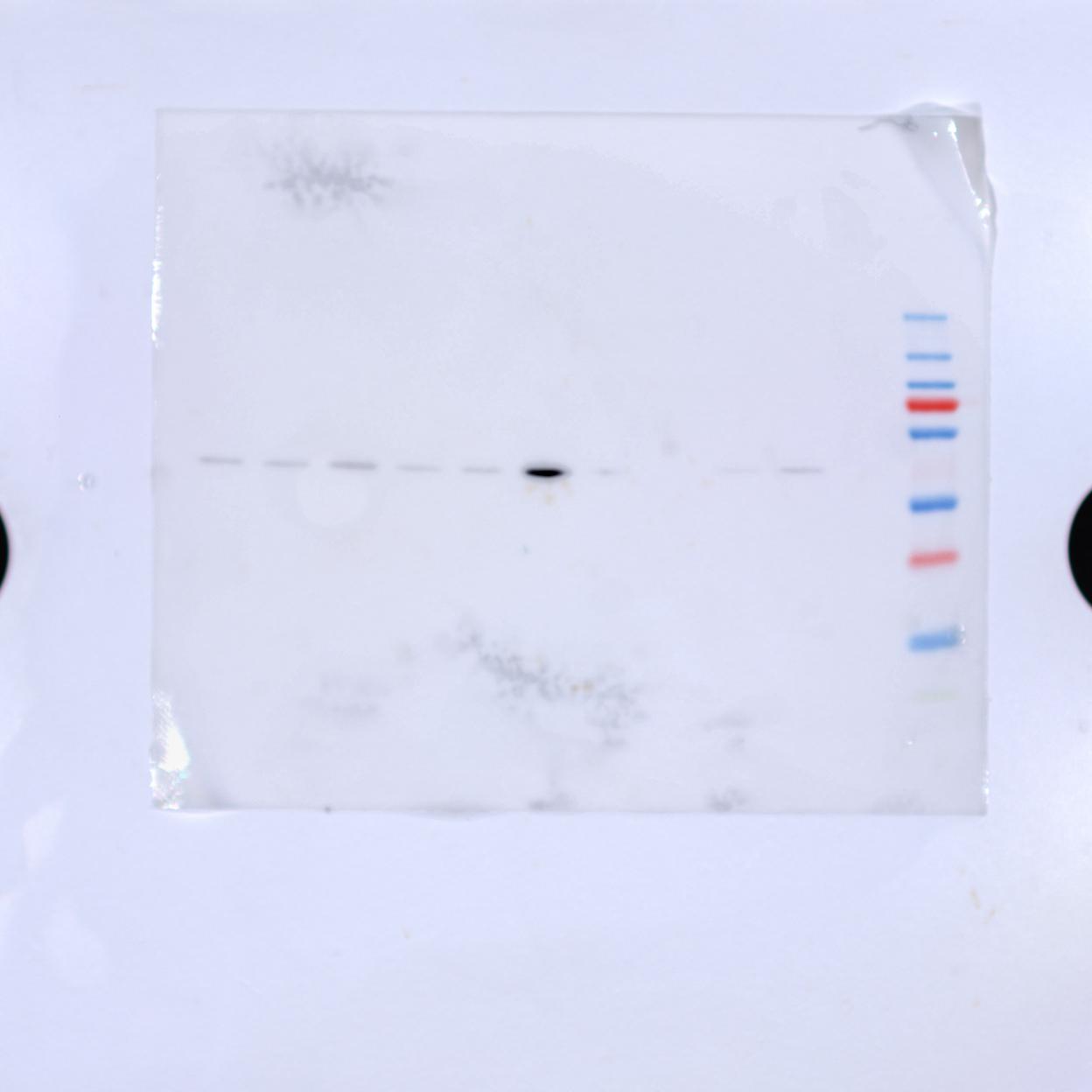

Supplement: Figure 3—source data 1. [file elife-53515-fig3-data1.zip › Source_data_Figure3/Figure3_panelD/20191101_145700_Ch_Fig3D_in_manuscript/20191101_145700_Ch+Marker.jpg]

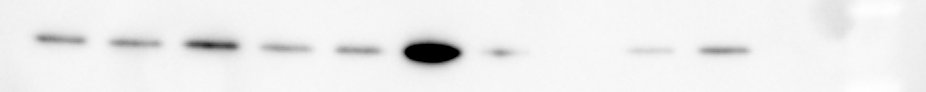

Supplement: Figure 3—source data 1. [file elife-53515-fig3-data1.zip › Source_data_Figure3/Figure3_panelD/20191101_145700_Ch_Fig3D_in_manuscript/Cropped_images/20191101_145700_Ch-1.jpg]

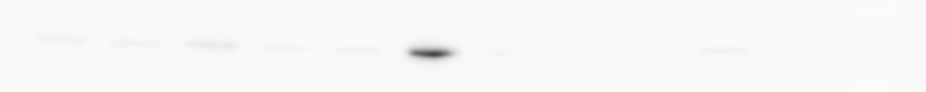

Supplement: Figure 3—source data 1. [file elife-53515-fig3-data1.zip › Source_data_Figure3/Figure3_panelD/20191101_145700_Ch_Fig3D_in_manuscript/Cropped_images/20191101_145700_Ch-1.tif]

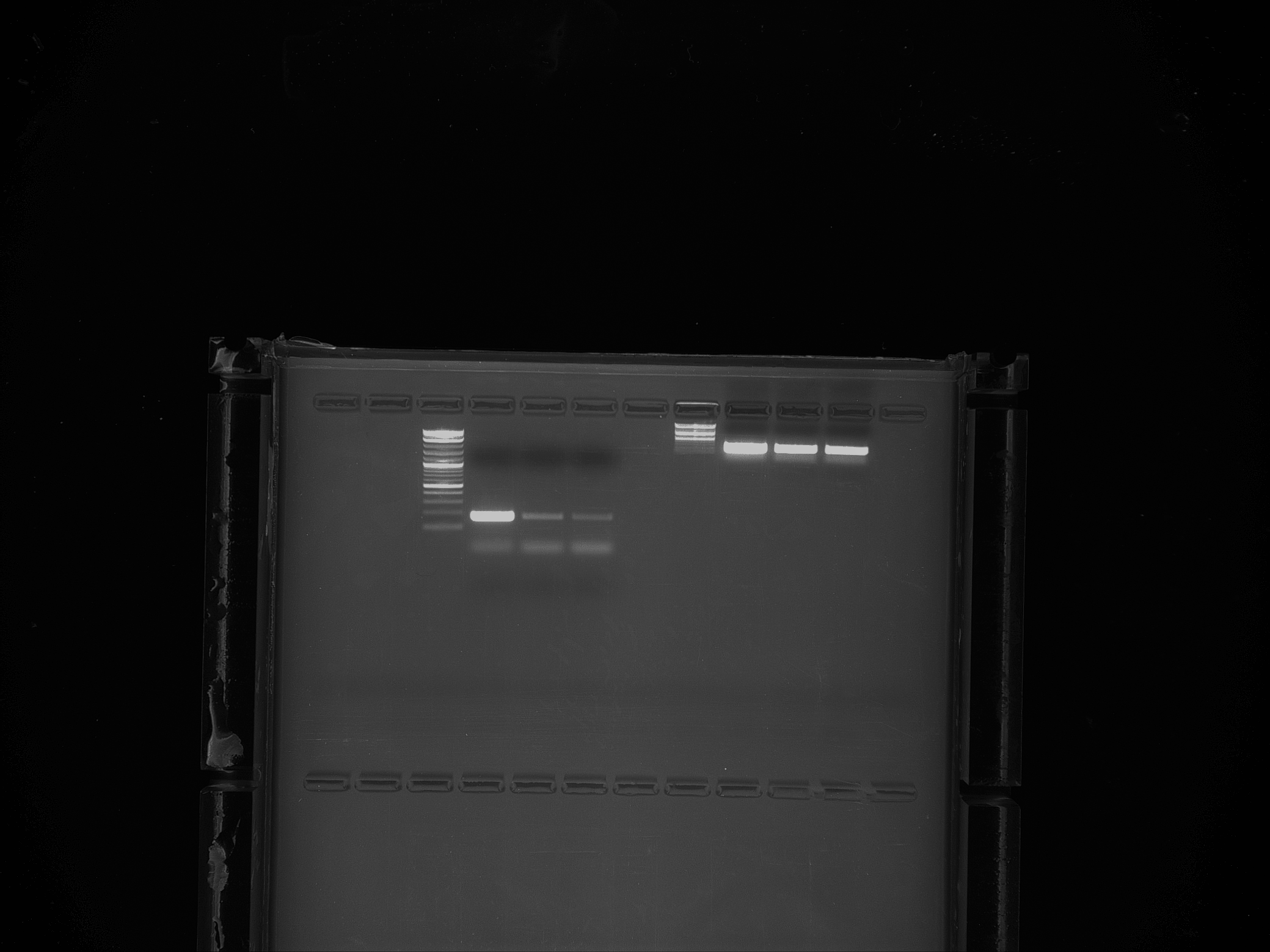

Supplement: Figure 3—figure supplement 1—source data 1. [file elife-53515-fig3-figsupp1-data1.zip › Source_data_Figure3_figure_supplement1/IM000219_169doubleparSbiotin_digestion_in_manuscript_uncropped.Tif]

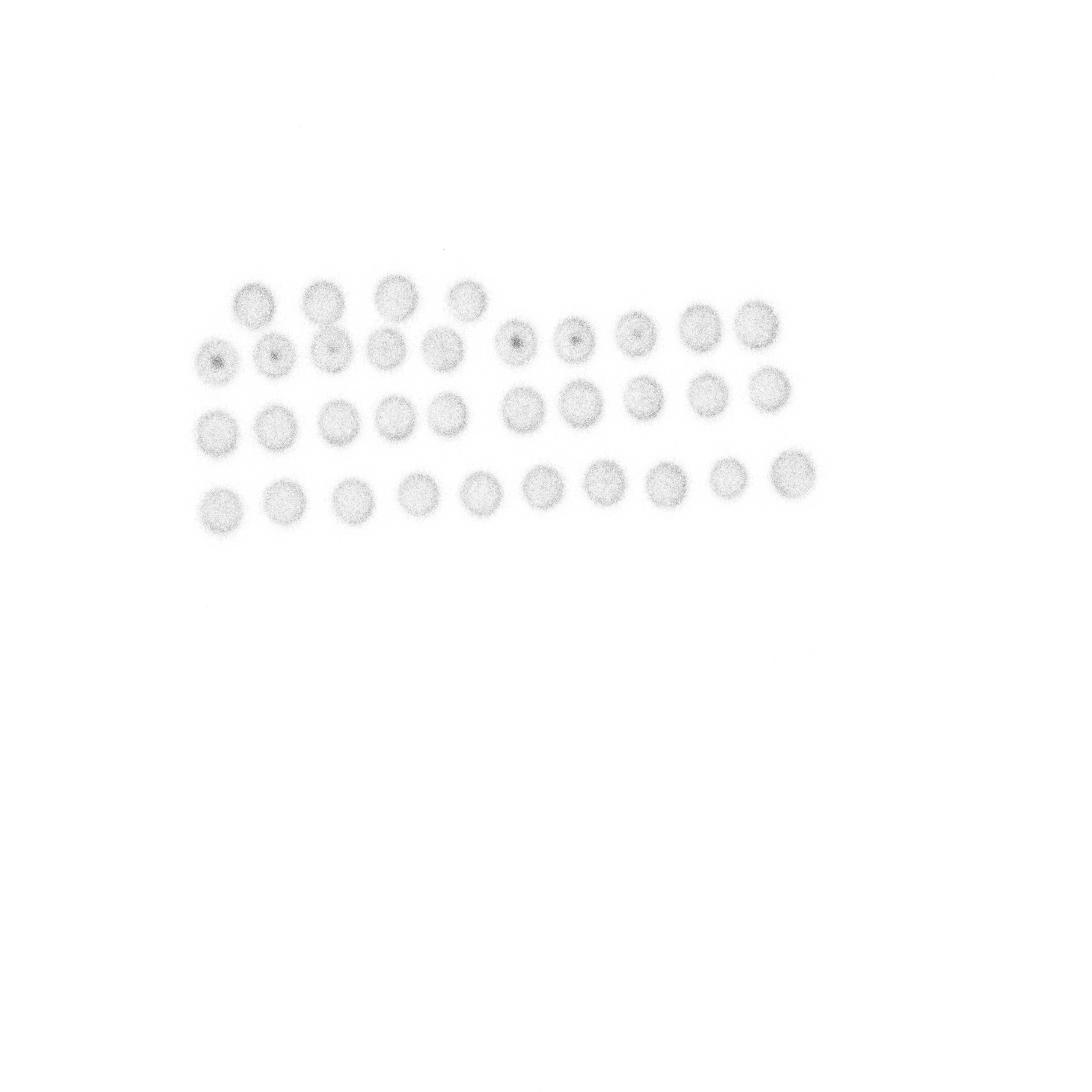

Supplement: Figure 5—source data 1. [file elife-53515-fig5-data1.zip › Source_data_Figure5/Figure5_panelA/dracala 3010_Fig5A_and_repeats_in_manuscript_uncropped.tif]

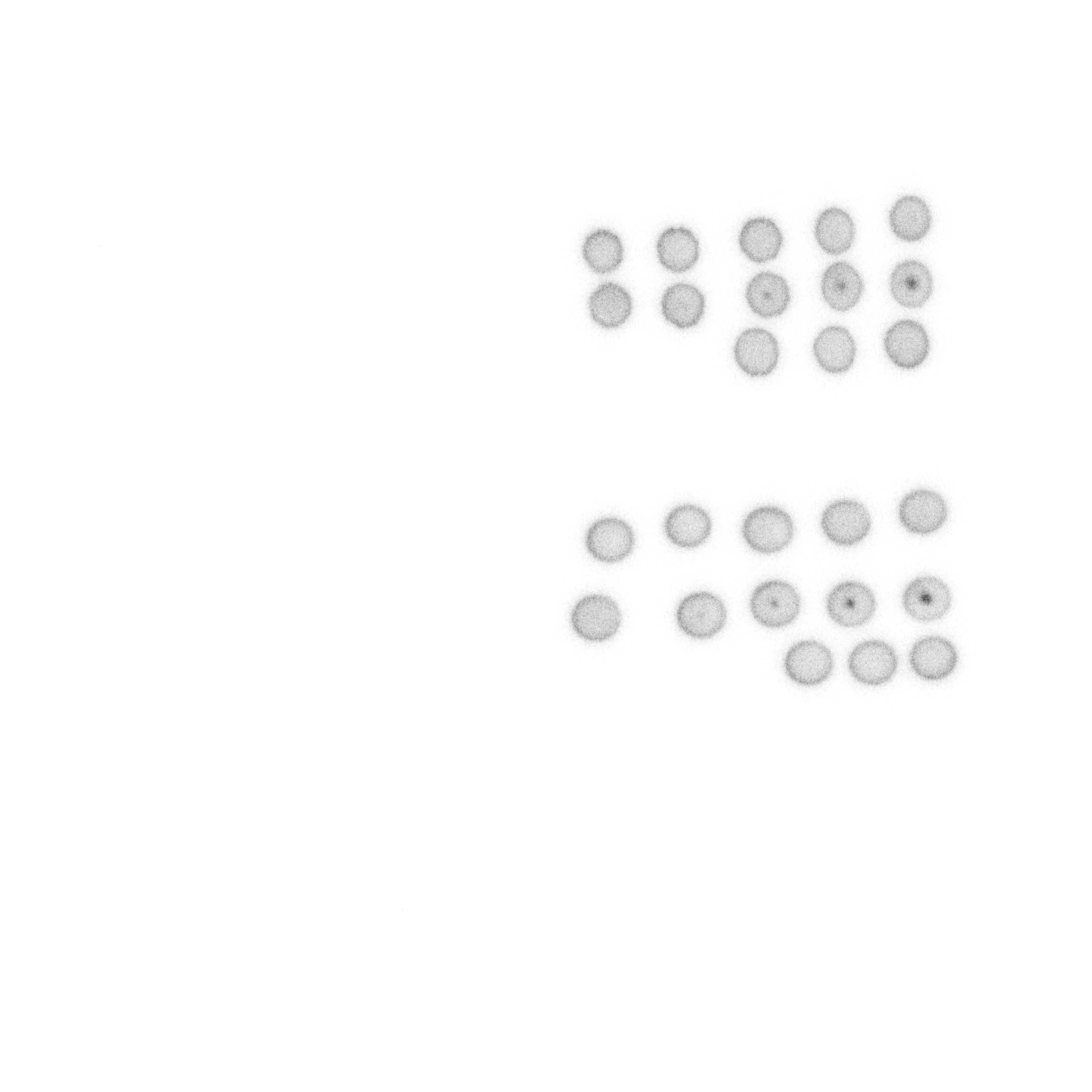

Supplement: Figure 5—source data 1. [file elife-53515-fig5-data1.zip › Source_data_Figure5/Figure5_panelA/dracala 3010_Fig5A_other_repeats_uncropped.tif]

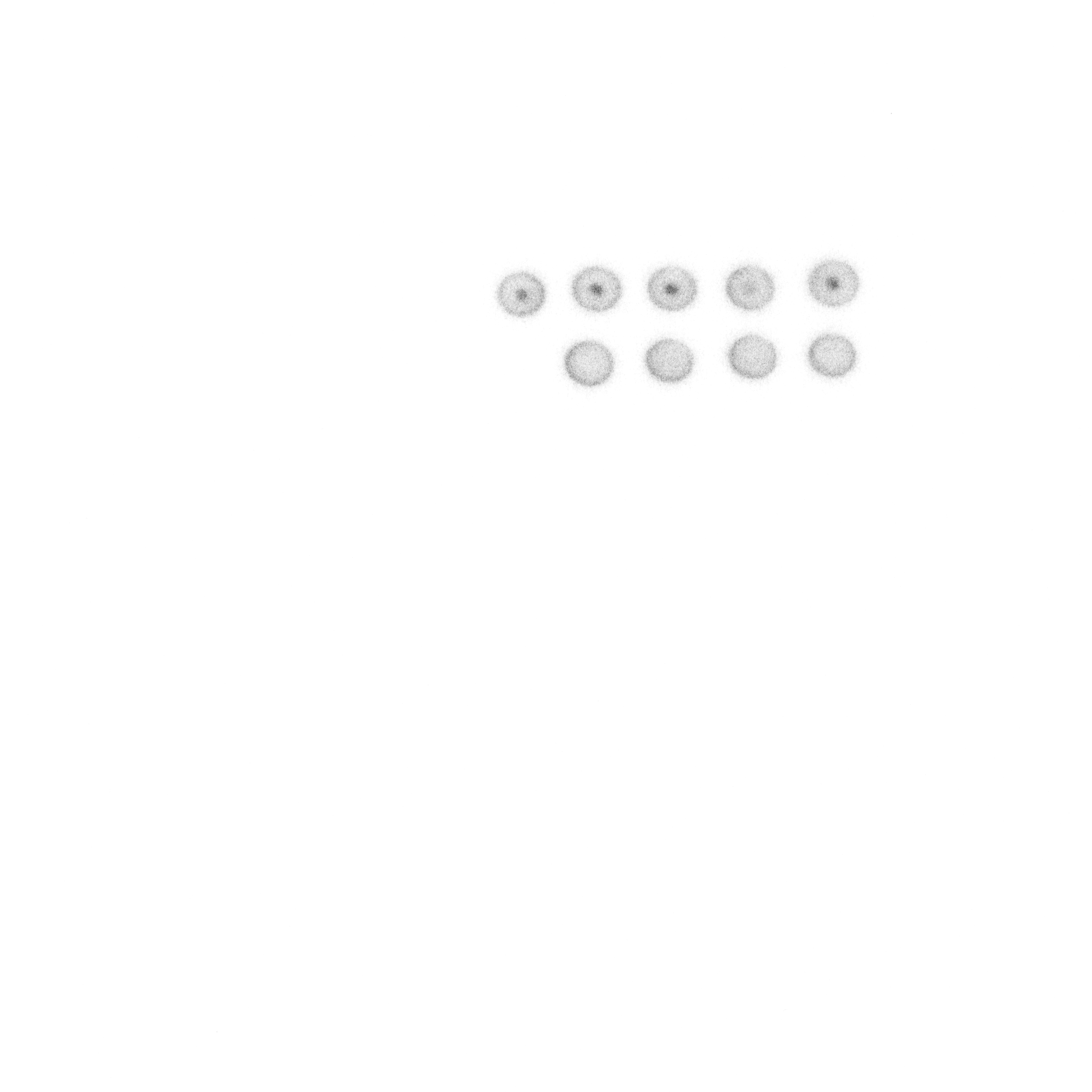

Supplement: Figure 5—source data 1. [file elife-53515-fig5-data1.zip › Source_data_Figure5/Figure5_panelB/dracala 2810-9_Fig5B_other_repeats_uncropped.tif]

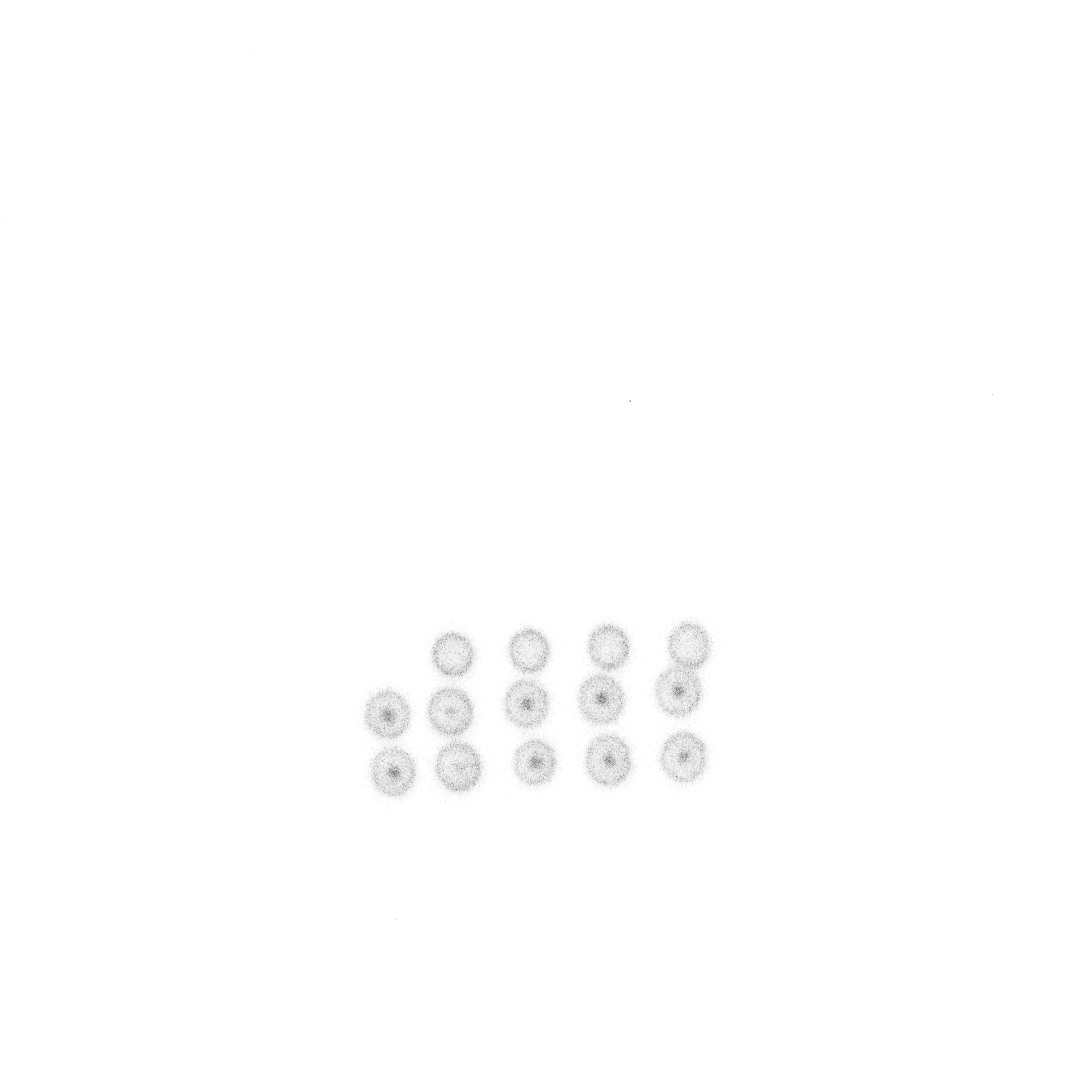

Supplement: Figure 5—source data 1. [file elife-53515-fig5-data1.zip › Source_data_Figure5/Figure5_panelB/dracala 3010-1_Fig5B_and_repeats_in_manuscript_uncropped.tif]

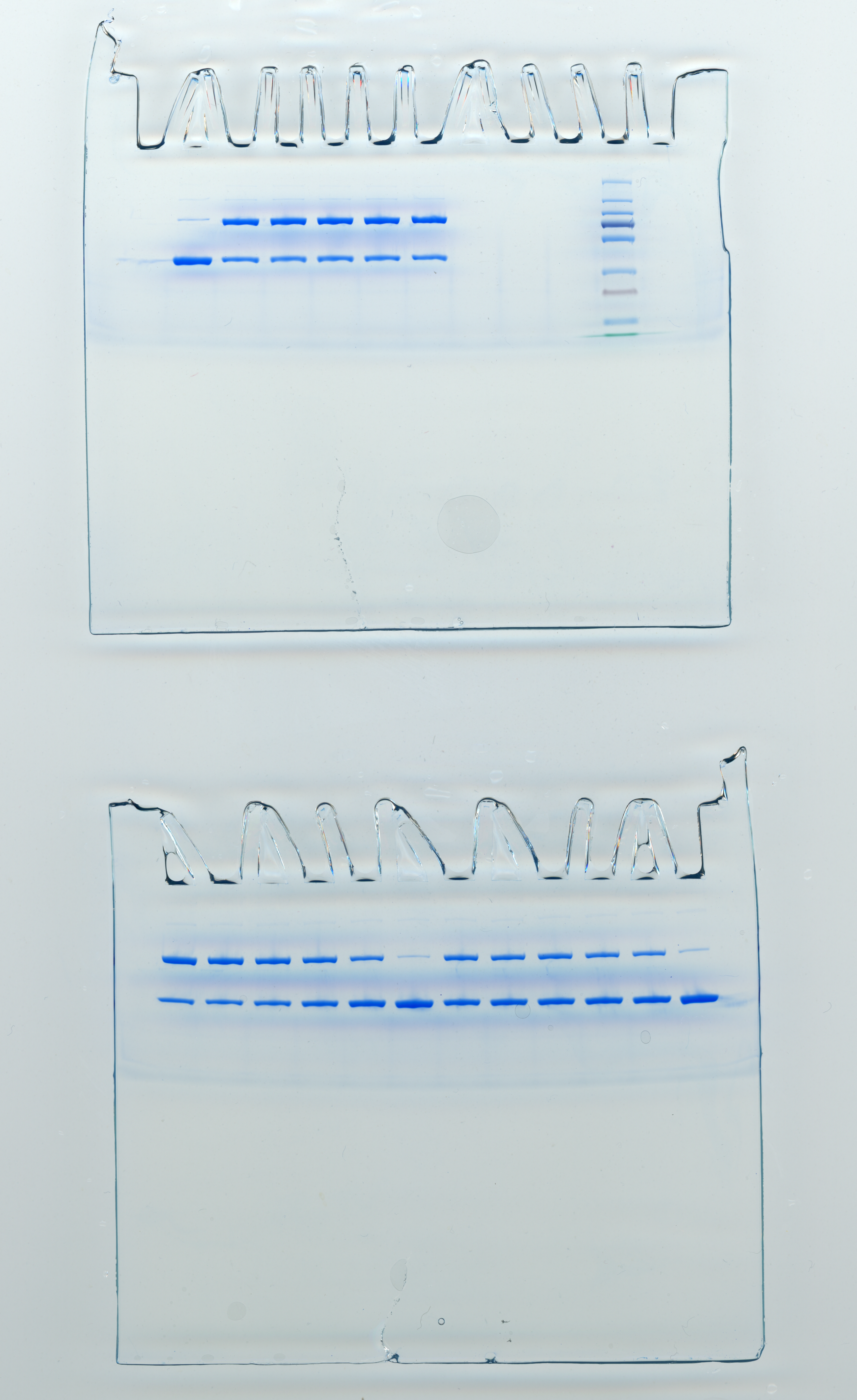

Supplement: Figure 6—source data 1. [file elife-53515-fig6-data1.zip › Source_data_Figure6/Source_data_Figure6/Figure6_panelB/time_course_X_link_Figure6B_001_uncropped.tif]

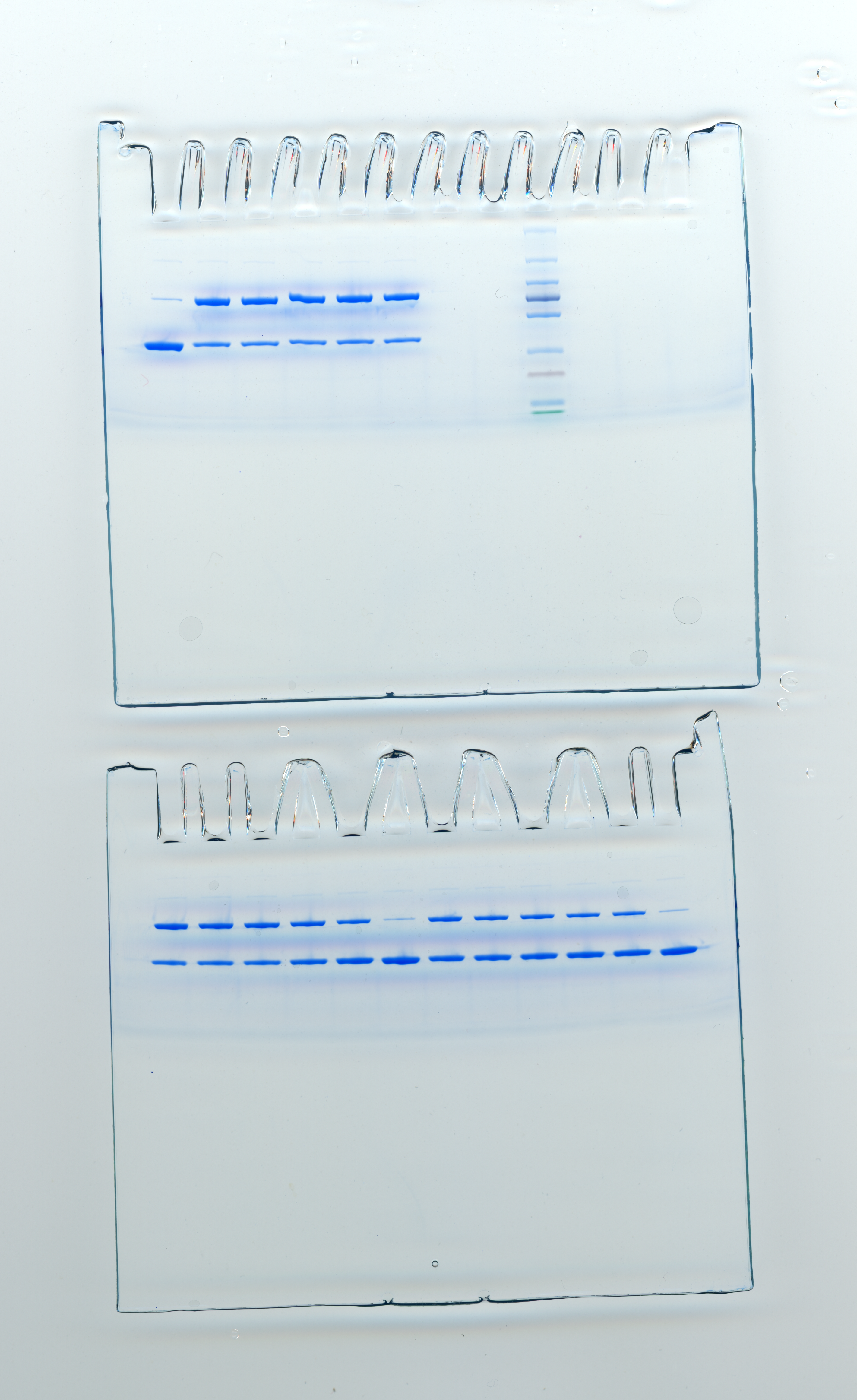

Supplement: Figure 6—source data 1. [file elife-53515-fig6-data1.zip › Source_data_Figure6/Source_data_Figure6/Figure6_panelB/time_course_X_link_Figure6B_002_uncropped.tif]

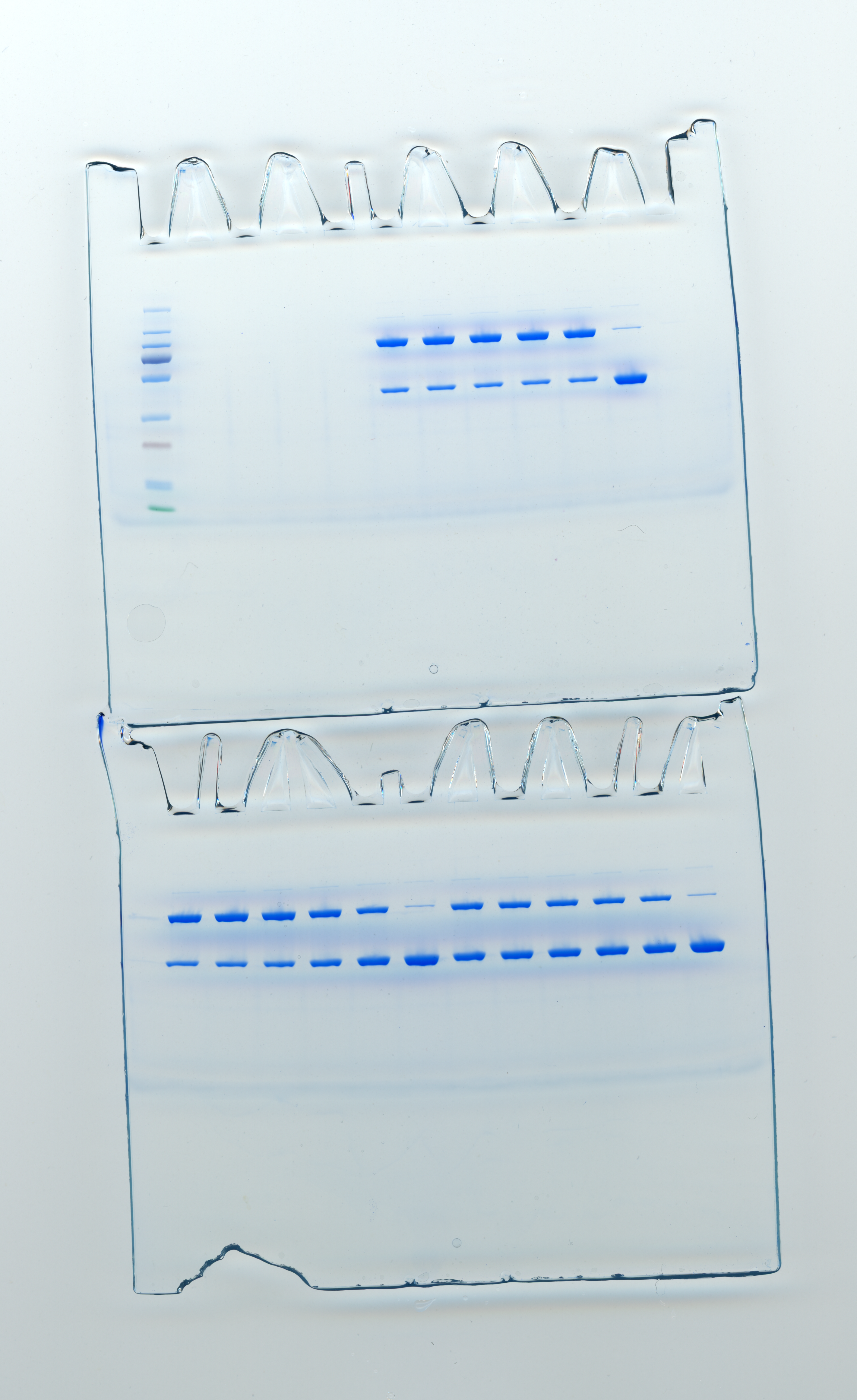

Supplement: Figure 6—source data 1. [file elife-53515-fig6-data1.zip › Source_data_Figure6/Source_data_Figure6/Figure6_panelB/time_course_X_link_Figure6B_003_uncropped.tif]

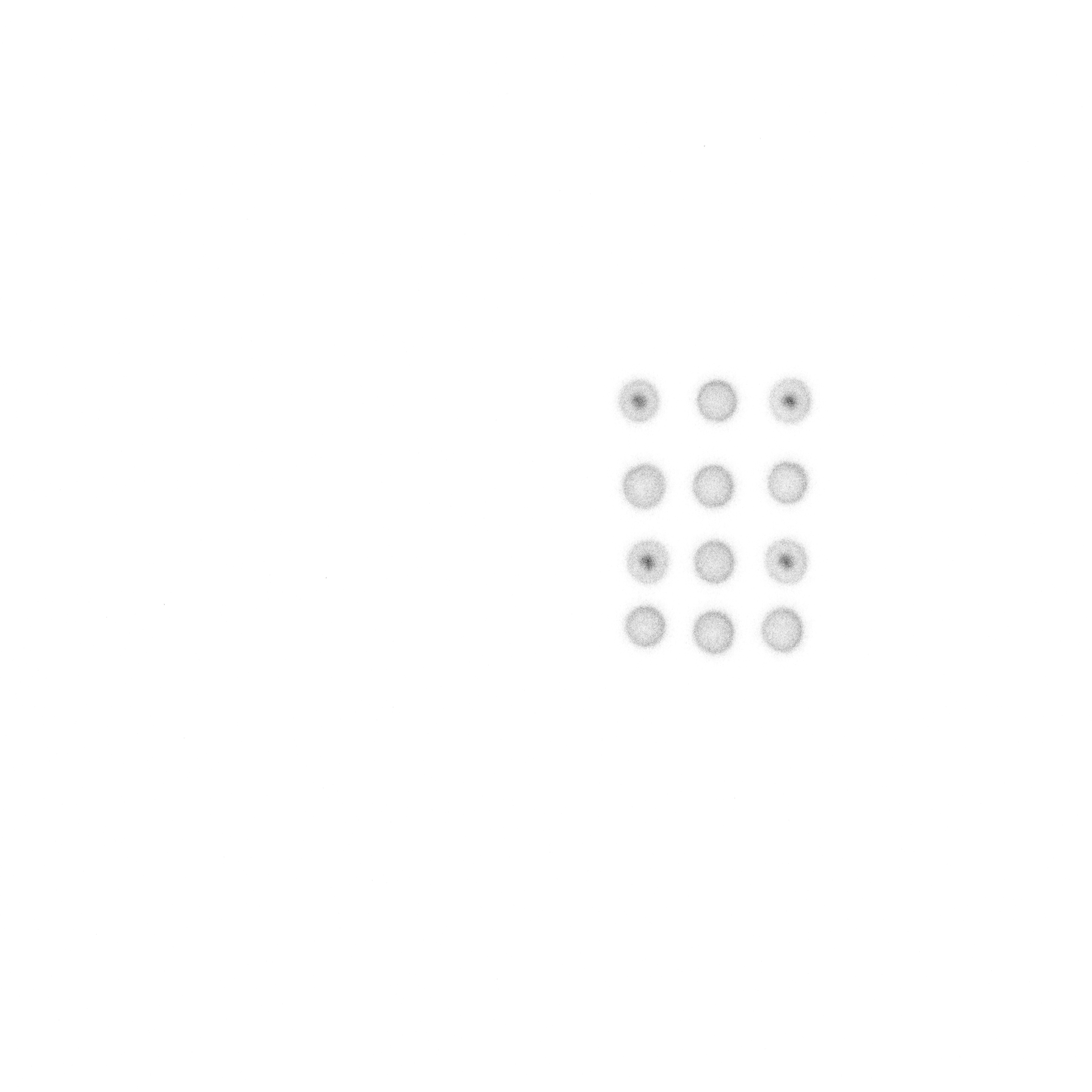

Supplement: Figure 6—figure supplement 1—source data 1. [file elife-53515-fig6-figsupp1-data1.zip › Source_data_Figure6_figure_supplement1/dracala_CTPgammaS_Fig6_fig_supplement1_other_repeats_uncropped.tif]

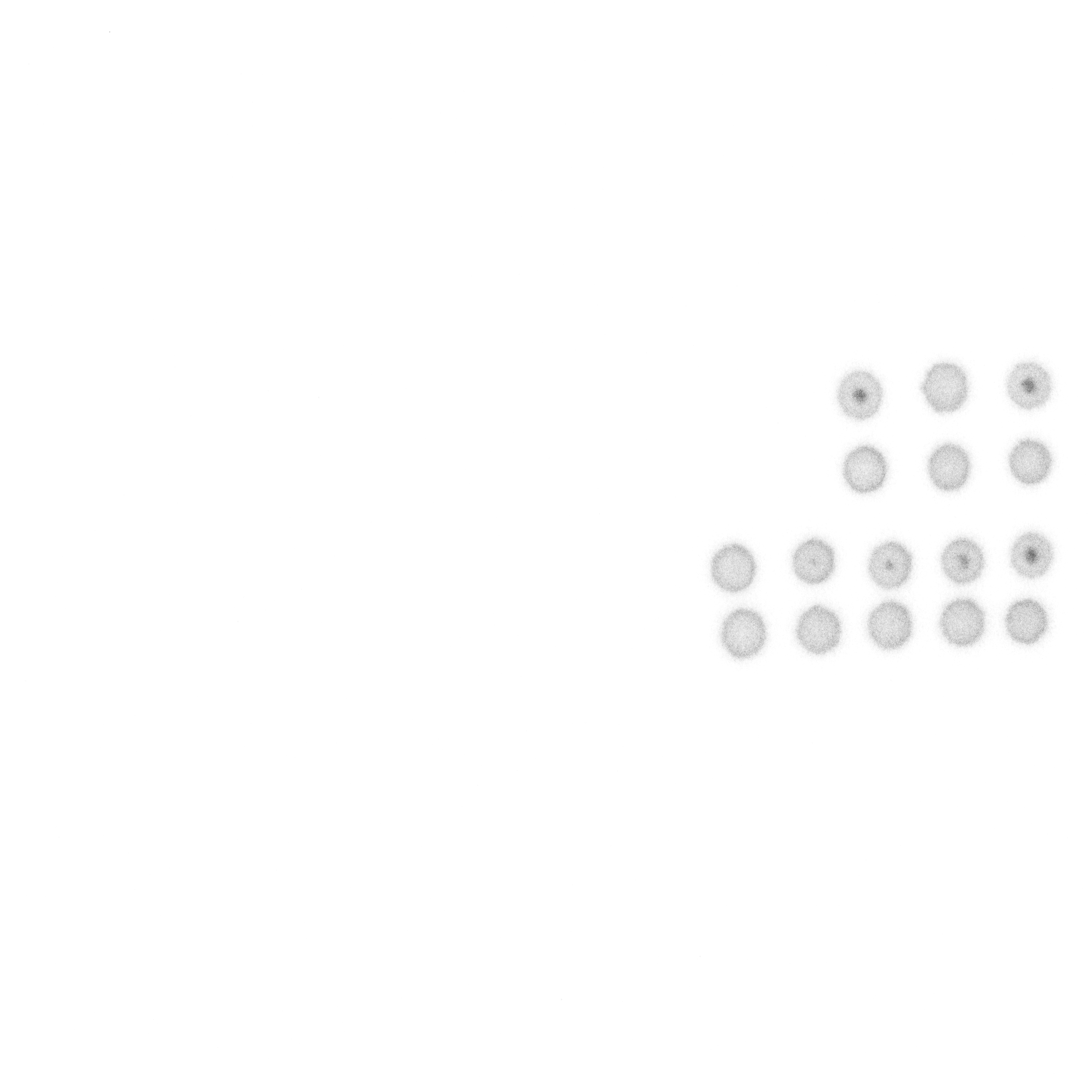

Supplement: Figure 6—figure supplement 1—source data 1. [file elife-53515-fig6-figsupp1-data1.zip › Source_data_Figure6_figure_supplement1/dracala181119-1_CTPgammaS_Fig6_fig_supplement1_in_manuscript_uncropped.tif]

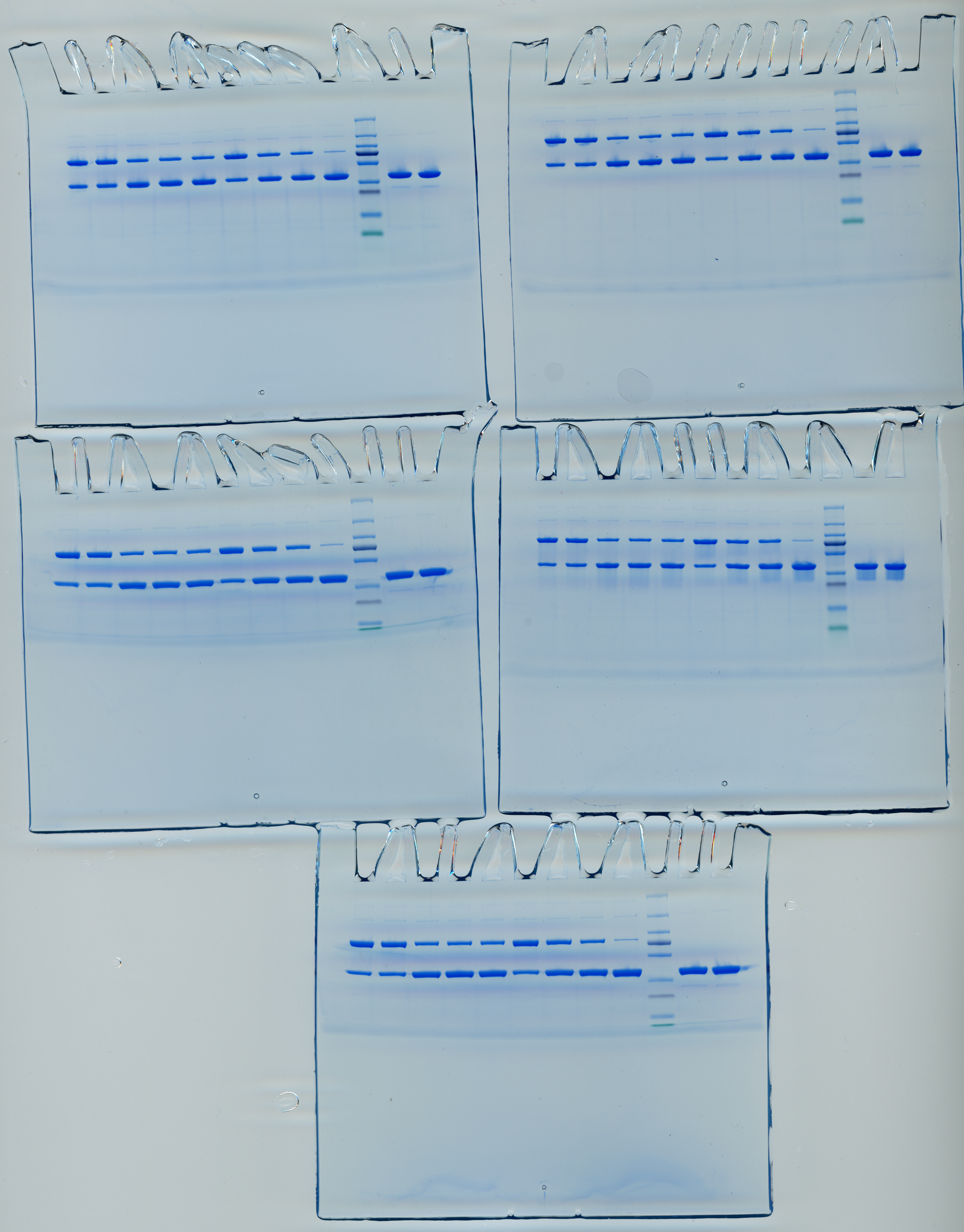

Supplement: Figure 6—figure supplement 2—source data 1. [file elife-53515-fig6-figsupp2-data1.zip › Source_data_Figure6_figure_supplement2/Figure6_figure_supplement2_panelB/Figure6_figure_supplement2B_uncropped.tif]
