# Supplementary material for: ParB spreading on DNA requires cytidine triphosphate in vitro
Source: eLife. 2020 Feb 20;9:e53515. doi: 10.7554/eLife.53515 (PMC7053999; doi:10.7554/eLife.53515)
Supplement: Supplementary file 1. [file elife-53515-supp1.docx]

**SUPPLEMENTARY FILE 1. PLASMIDS, DNA, AND PROTEIN SEQUENCES**

| **Plasmids/DNA** | **Description** | **Source** |
| --- | --- | --- |
| pET21b::*Caulobacter* ParB-His_6_ | Overexpression of C-terminally His_6_-tagged *Caulobacter* ParB, carbenicillin^R^  >*Caulobacter* ParB-His_6_  MSEGRRGLGRGLSALLGEVDAAPA**Q^35^**APGEQLGGSREAPIEILQRNPDQ  PRRTFREEDLEDLSNSIREKGVLQPILVRPSPDTAGEYQIVAGER**R^104^**WRA  AQRAGLKTVPIMVRELDDLAVLEIGIIENVQRADLNVLEEALSYKVLMEKF  ERTQENIAQTIGKSRSHVANTMRLLALPDEVQSYLVSGELTAGHARAIAA  AADPVALAKQIIEGGLSVRETEALARKAPNLSAGKSKGGRPPRVKDTDT  QALESDLSSVLGLDVSIDHRGSTGTLTITYATLEQLDDL**C^297^**NRLTRGIKLAA  ALEHHHHHH* (numbering according to ^1^) | Gift from C. Jacob-Wagner ^2^ |
| pET21b::*Caulobacter* ParB-His_6_ (C297S) | Overexpression of C-terminally His_6_-tagged *Caulobacter* ParB (C297S), carbenicillin^R^ | This study |
| pET21b::*Caulobacter* ParB-His_6_ (Q35C C297S) | Overexpression of C-terminally His_6_-tagged *Caulobacter* ParB (Q35C C297S), carbenicillin^R^ | This study |
| pET21b::TetR-His_6_ | Overexpression of C-terminally His_6_-tagged TetR (class B, from Tn10), carbenicillin^R^  >TetR (class B, from Tn10)-His_6_  MSRLDKSKVINSALELLNEVGIEGLTTRKLAQKLGVEQPTLYWHVKNKRALL  DALAIEMLDRHHTHFCPLEGESWQDFLRNNAKSFRCALLSHRDGAKVHL  GTRPTEKQYETLENQLAFLCQQGFSLENALYALSAVGHFTLGCVLEDQEH  QVAKEERETPTTDSMPPLLRQAIELFDHQGAEPAFLFGLELIICGLEKQLKC  ESGSKLAAALEHHHHHH* | This study |
| pUC19::260bp-*parS* | pUC19 plasmid with 260-bp insert that contains *parS* sites, carbenicillin^R^  >260-bp_natural_*Caulobacter*_*parS*_fragment_cloned_into_pUC19  caagacgctcgcctcaatgcgaacgcccccgggttcgagcgggggcg  ctggactcgatctatacgccaatcaggcgagcgggtcgatgtgactcatc  ggcgtttcacgtgaaacacccccaccgcagctgtgagcggcctgtggac  aatattggggatgttccacgtgaaacatcacttgccgatacagaaggtcg  aaaagacccgtccaagaacgtcctcaggatcgatacggccggagatg  cgctccagggcccgggc | This study |
| pUC19::260bp-scrambled *parS* | pUC19 plasmid with 260-bp insert that contains scrambled *parS* sites, carbenicillin^R^  >260-bp_scrambled_*Caulobacter*_*parS*_fragment_cloned_into_pUC19  caagacgctcgcctcaatgcgaacgcccccgggttcgagcgggggcg  ctggactcgatctatacgccaatcaggcgagcgggtcgatgtgactcatc  ggacagctcgagattcatcccccaccgcagctgtgagcggcctgtggac  aatattggggaatcgagtatacgctactcacttgccgatacagaaggtcg  aaaagacccgtccaagaacgtcctcaggatcgatacggccggagatg  cgctccagggcccgggc | This study |
| pET-His-MBP-TEV-DEST::*Sinorhizobium meliloti* ParB | For the purification of *Sinorhizobium meliloti* His_6_-MBP-ParB | ^3^ |
| pET-His-MBP-TEV-DEST::*Rhodobacter sphaeroides* ParB | For the purification of *Rhodobacter sphaeroides* His_6_-MBP-ParB | This study |
| pET-His-MBP-TEV-DEST::*Thermus thermophilus* ParB | For the purification of *Thermus thermophilus* His_6_-MBP-ParB | ^3^ |
| pET-His-MBP-TEV-DEST::*Dechloromonas aromatica* ParB | For the purification of *Dechloromonas aromatica* His_6_-MBP-ParB | This study |
| pET-His-MBP-TEV-DEST::*Psychrobacter* spp. ParB | For the purification of *Psychrobacter* spp. His_6_-MBP-ParB | This study |
| pET-His-MBP-TEV-DEST::*Staphylococcus aureus* ParB | For the purification of *Staphylococcus aureus* His_6_-MBP-ParB | ^3^ |
| pET-His-MBP-TEV-DEST::*Zymomonas mobilis* ParB | For the purification of *Zymomonas mobilis* His_6_-MBP-ParB | This study |
| pET-His-MBP-TEV-DEST::*Xanthomonas campestris* ParB | For the purification of *Xanthomonas campestris* His_6_-MBP-ParB | ^3^ |
| 169bp_*parS* | cgccagggttttcccagtcacgacgttgtaaaacgacggccagtgaattcgagctcggtac  ccgcaggaggacgtagggtaggggga**tgtttcacgtgaaaca**ggggatcctctagagtc  gacctgcaggcatgcaagcttggcgtaatcatggtcatagctgtttcct | This study |
| 169bp_scrambled_*parS* | cgccagggttttcccagtcacgacgttgtaaaacgacggccagtgaattcgagctcggtacc  cgcaggaggacgtagggtaggggga***aattacactgagttta***ggggatcctctagagtcga  cctgcaggcatgcaagcttggcgtaatcatggtcatagctgtttcct | This study |
| 170bp_*parS* | cgccagggttttcccagtcacgacgttgtaaaacgacggccagaattcgcaacgtg  **tgtttcacgtgaaaca**gccttgaactgataacgactctatcattgatagagtgttctct  ccacgggatccccaggcatgcaagcttggcgtaatcatggtcatagctgtttcct | This study |
| around_pUC19_F | tcactcatggttatggcagcactgcataattc | This study |
| around_pUC19_F | taacactgcggccaacttacttctgacaacg | This study |
| 20bp_*parS*_BLI_probeF | [Biotin]GGGAtgTTTCACGTGAAAca | This study |
| 20bp_*parS*_BLI_probeR | tgTTTCACGTGAAAcaTCCC | This study |
| 28bp_*tetO*_BLI_probeF | [Biotin]ggggactctatcattgatagagtatgc | This study |
| 28bp_*tetO*_BLI_probeR | gcatactctatcaatgatagagtcccc | This study |
| 20bp_*NBS*_BLI_probeF | [Biotin]GGGAtaTTTCCCGGGAAAta | This study |
| 20bp_*NBS*_BLI_probeR | taTTTCCCGGGAAAtaTCCC | This study |

**Keys:**

M13F (-47): cgccagggttttcccagtcacgac M13R: aggaaacagctatgaccat

*parS:* **tgtttcacgtgaaaca** scrambled *parS*: ***aattacactgagttta***

*tetO:* actctatcattgatagagt *Bam*HI RS: ggatcc *Eco*RI RS: gaattc

**SUPPLEMENTARY REFERENCES**

1. Tran, N. T. *et al.* Permissive zones for the centromere-binding protein ParB on the Caulobacter crescentus chromosome. *Nucleic Acids Res* **46**, 1196–1209 (2018).

2. Lim, H. C. *et al.* Evidence for a DNA-relay mechanism in ParABS-mediated chromosome segregation. *Elife* **3**, e02758 (2014).

3. Jalal, A. S. B. *et al.* Evolving a new protein-DNA interface via sequential introduction of permissive and specificity-switching mutations. *bioRxiv* 724823 (2019) doi:10.1101/724823.
